# A spurious correlation between difference scores in evidence-accumulation model parameters

**DOI:** 10.3758/s13428-022-01956-8

**Published:** 2022-09-22

**Authors:** James A. Grange, Stefanie Schuch

**Affiliations:** 1 Keele University, Keele, UK; 2 RWTH Aachen University, Aachen, Germany

**Keywords:** Diffusion modelling, Individual differences, RT difference scores, Correlations

## Abstract

Evidence-accumulation models are a useful tool for investigating the cognitive processes that give rise to behavioural data patterns in reaction times (RTs) and error rates. In their simplest form, evidence-accumulation models include three parameters: The average rate of evidence accumulation over time (drift rate) and the amount of evidence that needs to be accumulated before a response becomes selected (boundary) both characterise the response-selection process; a third parameter summarises all processes before and after the response-selection process (non-decision time). Researchers often compute experimental effects as simple difference scores between two within-subject conditions and such difference scores can also be computed on model parameters. In the present paper, we report spurious correlations between such model parameter difference scores, both in empirical data and in computer simulations. The most pronounced spurious effect is a negative correlation between boundary difference and non-decision difference, which amounts to *r* = – .70 or larger. In the simulations, we only observed this spurious negative correlation when either (a) there was no true difference in model parameters between simulated experimental conditions, or (b) only drift rate was manipulated between simulated experimental conditions; when a true difference existed in boundary separation, non-decision time, or all three main parameters, the correlation disappeared. We suggest that care should be taken when using evidence-accumulation model difference scores for correlational approaches because the parameter difference scores can correlate in the absence of any true inter-individual differences at the population level.

**Supplementary Information:**

The online version contains supplementary material available at 10.3758/s13428-022-01956-8.

In recent years, renewed attempts have been made to bridge the long-recognized gap between experimental cognitive psychology on one hand, and inter-individual differences research on the other (e.g., Borsboom et al., 2009; Cronbach, 1957; Euler & Schubert, 2021; Hedge et al., 2017; Miller & Ulrich, 2013; Parsons et al., 2019; Rouder et al., 2019; Rouder & Haaf, 2019). However, these two branches of psychological research use different methodologies, making it difficult to bridge the gap.

One problem is the so-called “reliability paradox” (Hedge et al., 2017): It has repeatedly been observed that standard experimental effects such as the Stroop effect, the Simon effect, or the task-switch cost—effects that have been replicated in thousands of studies—have surprisingly low split-half and retest reliability. Moreover, when the same effect is measured as response time (RT) difference score and error difference score, even these two measures of the same effect often do not correlate (e.g., Hedge et al., 2018). Part of the problem is that experimental psychology and inter-individual differences psychology focus on two different kinds of reliability: Experimental psychology aims to provide effects that occur in all (or almost all) individuals and are of similar size in all individuals, and therefore are replicable in group-level analyses across different samples. In contrast, psychological research into inter-individual differences looks for effects that consistently and reliably differ across individuals (see also Rouder & Haaf, 2019 for a discussion of this distinction). Here, an effect is reliable when the same rank-ordering of individuals can be reproduced across different data sets from the same group of individuals. Hence, reliability in the sense of experimental research and reliability in the psychometric sense are two different concepts that are not easily reconciled.

## Evidence-accumulation models

**Fig. 1:**
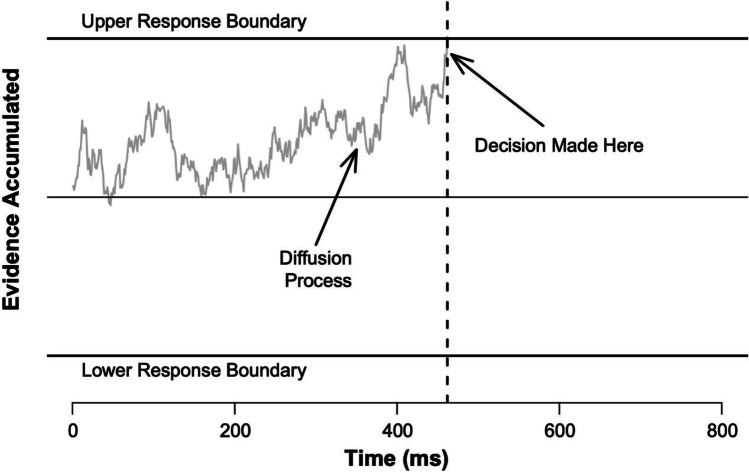
Schematic representation of trial processing in the drift-diffusion model. Note some researchers refer to the response boundary as an “evidence threshold.” Fig. available at https://www.flickr.com/photos/150716232@N04/46893547582 under CC license https://creativecommons.org/licenses/by/2.0/

As a possible way of increasing the reliability of experimental effects in the psychometric sense, researchers have started to use formal computational modelling, such as evidence-accumulation models, taking model parameters instead of behavioural measures (such as mean RT and mean error rates) as primary dependent variables (e.g., Lerche & Voss, 2017; Lerche et al., 2020; Hedge et al., 2018, 2019, 2021; Ratcliff & Childers, 2015; Schubert et al., 2015, 2016, 2021; for using evidence-accumulation models for correlational approaches in cognitive neuroscience, see e.g., Forstmann et al., 2011, 2016). Evidence-accumulation models are a class of formal computational models that can be applied to speeded choice RT tasks with two or more response alternatives. Such models assume that evidence for the different response alternatives is accumulated over time until an evidence threshold/boundary for one response alternative is reached; this response alternative then becomes selected. In their simplest form, these models include two parameters for the response-selection process: the average rate of evidence accumulation over time (drift rate), and the height of the threshold/boundary that needs to be reached (boundary separation). A third parameter in these models summarises all processes before and after the response-selection process and is often called non-decision time. Prominent examples of evidence-accumulation models are the drift-diffusion model (DDM) introduced by Ratcliff and colleagues (e.g., Ratcliff & McKoon, 2008; Ratcliff et al., 2016; see Fig. 1), and the Linear Ballistic Accumulator (LBA) model by Heathcote and Brown (2008). More complex models have been developed that include additional parameters (see, e.g., Servant et al., 2014; White et al., 2018, for discussion and comparison of models designed to account for performance in conflict tasks).

Evidence-accumulation models can be useful for a better understanding of the relationship between experimental psychology and interindividual-differences research. For instance, Hedge et al. (2018) investigated the surprising lack of a consistent correlation between RT difference scores and error difference scores of the same effect using evidence-accumulation models. They demonstrated that this lack of correlation between behavioural difference scores of the same effect can be understood when considering difference scores in DDM parameters. In general, the experimental effect (be it the Stroop effect, Simon effect, task-switch cost, or other) is computed as the within-subject difference between two conditions, which can be termed the “easy” and “hard” conditions for sake of generality. Hard-minus-easy difference scores can be computed on behavioural measures as well as on model parameter values. If participants differ in the size of their difference score in the evidence-accumulation parameter, then RT difference scores and error difference scores will be *positively* correlated across participants. If, in addition, participants differ in their overall threshold setting, the same difference in evidence accumulation between hard and easy conditions will produce larger RT effects—and at the same time smaller error effects—in those individuals with higher thresholds than in those individuals with lower thresholds. This latter effect can produce a negative, or close-to-zero, correlation between RT difference scores and error difference scores across individuals. Hence, relying on behavioural difference scores alone does not make much sense for correlational approaches because correlations between behavioural difference scores can be positive, negative, or non-existent, depending on the underlying cognitive processes that cause the interindividual variability in the behavioural difference scores.

In light of these insights, it may be tempting to rely more on model-parameter difference scores instead of behavioural difference scores. In fact, in experimental psychology, several researchers have investigated common experimental effects with simple evidence-accumulation models, and computed model-parameter difference scores, for example for the response-effect compatibility effect (Janczyk & Lerche, 2019), the backward-crosstalk effect in dual-tasking (Durst & Janczyk, 2019; Janczyk et al., 2017); task-switch costs (Schmitz & Voss, 2012, 2014); N–2 task-repetition costs in task switching (Kowalczyk & Grange, 2020; Schuch, 2016; Schuch & Grange, 2019; Schuch & Konrad, 2017) and speed–accuracy trade-off effects (Forstmann et al., 2011; see Heitz, 2014, for a review).

Such difference scores in model parameters obtained from experimental psychology could in turn be used for correlational approaches, potentially yielding more reliable correlations. Thus, difference scores in model parameters could be a promising tool for integrating cognitive experimental psychology and inter-individual differences research.

## Overview of the present paper

Regarding the possibility of using difference scores from evidence-accumulation model parameters for investigating inter-individual differences, in the present paper we highlight a potential problem with such model-based difference scores. We incidentally observed a strong negative correlation between two parameters of a simple evidence-accumulation model in our own data sets and set out to investigate this finding in a more systematic way. In particular, when applying a simple drift-diffusion model, we observed a pronounced correlation between the boundary separation parameter difference score and the non-decision time parameter difference score: Those participants who show a higher boundary in the hard than easy condition typically show a smaller non-decision time in the hard than easy condition, and vice versa. This incidental finding raised our curiosity, and we checked several more of our own data sets. We observed a pronounced negative correlation in all of them of around *r* = – .70 between the difference scores in boundary separation parameter and the difference scores in non-decision time parameter across participants. We also checked whether this negative correlation was present in data collected independently of either of our research groups by reanalysing some of the data from Dutilh et al. (2019), and found it was indeed present.

This incidental finding made us wonder whether this is a theoretically interesting effect, or perhaps a methodological artefact of some sort. We therefore conducted a series of computational simulations where we knew the true population parameter values and could manipulate them systematically. The simulations revealed that even in the absence of any differences in population parameter values between the easy and hard conditions, a correlation of difference scores in DDM parameters emerged. The pronounced negative correlation consistently occurs between the difference score in boundary separation parameter and the difference score in non-decision time; at the same time, there are no such pronounced correlations of one of these difference scores with the difference score in the evidence-accumulation rate parameter (although we sometimes did observe a moderate positive correlation between boundary separation difference score and evidence-accumulation difference score).

Notably, the pronounced negative correlation in the simulations is *only* observed between parameter *difference scores* of boundary separation and non-decision time, while at the same time, the overall values of boundary separation and non-decision time parameters within a condition are not highly correlated. That is, we observed a pronounced negative correlation between the hard-minus easy *difference score* in boundary separation parameter and the hard-minus-easy *difference score* in non-decision time parameter, while there are no such pronounced correlations between boundary separation parameter and non-decision time parameter within the hard condition, or within the easy condition.

Our further investigations revealed that this spurious correlation of model difference scores is not constricted to one particular kind of evidence-accumulation model, or to the fitting algorithms of one particular software package; rather, we observed the correlation both when using the Ratcliff drift-diffusion model and the Linear Ballistic Accumulator Model (Brown & Heathcote, 2008), and with different software packages (*fast-dm-30*: Voss et al., 2015; Voss & Voss, 2007; *EZ-diffusion model*: Wagenmakers et al., 2007; *rtdists*: Singmann et al., 2020).

Diffusion models fall within the class of so-called "sloppy" models where parameters are known to be highly correlated—a characteristic typical of many biologically plausible models—which can impact on the efficiency and accuracy of parameter estimation (Boehm et al., 2018; Heathcote et al., 2019). Indeed, recent studies have shown that when independent research teams attempt to fit versions of the diffusion model to a single data set, the teams often produce quite different estimates of best-fitting model parameters, and these differences sometime lead to different inferences (Boehm et al., 2018; Dutilh et al., 2019). Documenting the extent of these correlations is therefore of importance for researchers wishing to draw valid inferences from their modelling. Our findings imply that care should be taken when interpreting diffusion-model difference scores in boundary-separation parameter and non-decision time parameter. These difference scores can correlate in the absence of any population-level differences between two within-subjects experimental conditions. Moreover, the difference scores in these two model parameters should not be used in individual-differences research, because they do not necessarily reflect true inter-individual differences at the population level.

In the following, we will first describe the spurious correlation in two empirical data sets of previously published studies, one from our own labs (Schuch & Grange, 2019), and one from a different lab (Dutilh et al., 2019). Then we will describe in detail the set of computational simulations, and we will conclude with some considerations on what could cause the spurious correlation and some recommendations for researchers wishing to apply these models.

## Reanalysis of behavioural data

In several of our own data sets (from published and unpublished studies), we observed a pronounced negative correlation between participants’ boundary-separation parameter difference score and their non-decision time parameter difference score when applying a simple drift-diffusion model. In the following, we exemplarily present a reanalysis of a previously published study of ours (Schuch & Grange, 2019), where this correlation amounted to about *r* = – .70 in all experimental conditions.

## Schuch and Grange (2019)

In the Schuch and Grange’s (2019) study, N–2 task repetition costs were measured, which are a kind of task-switch cost where different types of task sequences are compared. N–2 task repetition costs denote the finding of worse performance in task sequences of the type ABA (where the task performed in trial N is the same as the task performed in trial N–2) as compared to type CBA (where the task in trial N is not the same as in trial N–2). The performance decrement in ABA versus CBA is usually taken as an indicator of persisting task-level inhibition (Mayr & Keele, 2000; see Gade et al., 2014; Koch et al., 2010; Mayr, 2007, for reviews). The reasoning is that task A becomes inhibited when switching from A to B, and this inhibition decays slowly over time; the sooner one switches back to the previously inhibited task, the more persisting inhibition needs to be overcome. While Schuch and Grange (2019) focused on a different research question (investigating aftereffects of N–2 repetition costs; not relevant for the present context), the data can be summarized as an assessment of N–2 repetition costs in four different experimental conditions. In particular, ABA and CBA trials were obtained in the experimental conditions I, II, III, and IV (condition I: short task-preparation interval and task sequence preceded by another ABA sequence; condition II: short task-preparation interval and task sequence preceded by another CBA sequence; condition III: long task-preparation interval and task sequence preceded by another ABA sequence; condition IV: long task-preparation interval and task sequence preceded by another CBA sequence). All independent variables were manipulated within-subjects, meaning that for each subject, four different N–2 repetition costs (calculated as the difference score of ABA minus CBA) can be computed (one for each experimental condition I, II, III, and IV). From here we refer to this as the “difference score.” We took the diffusion modelling results from Schuch and Grange (2019), and correlated the difference score in boundary separation parameter with the difference score in non-decision time across participants, separately for each condition I, II, III, and IV.

The DDM analysis in the Schuch and Grange (2019) study was conducted with the software “*fast-dm-30*” (Voss et al., 2015). The parameters drift rate (*v*)*,* boundary separation (*a*)*,* non-decision time (*t0*), and trial-by-trial variability of non-decision time (*st0*) were estimated separately for each individual and each condition. The starting point bias was set to 0.5*a* (i.e., in the middle between the two boundaries). The lower and upper boundaries were set to reflect correct and wrong responses, respectively. All other parameters implemented in *fast-dm* were set to 0. The Kolmogorov–Smirnov (KS) statistic was used for fitting.

From here onwards, we use the labels *v, a*, and *t0* to refer to the three main diffusion model parameters drift rate, boundary separation, and non-decision time respectively, and we use the terms *v-*difference, *a*-difference, and *t0*-difference, to refer to the parameter difference scores between two within-subject conditions.

### Experiment 1 from Schuch and Grange (2019)

**Fig. 2:**
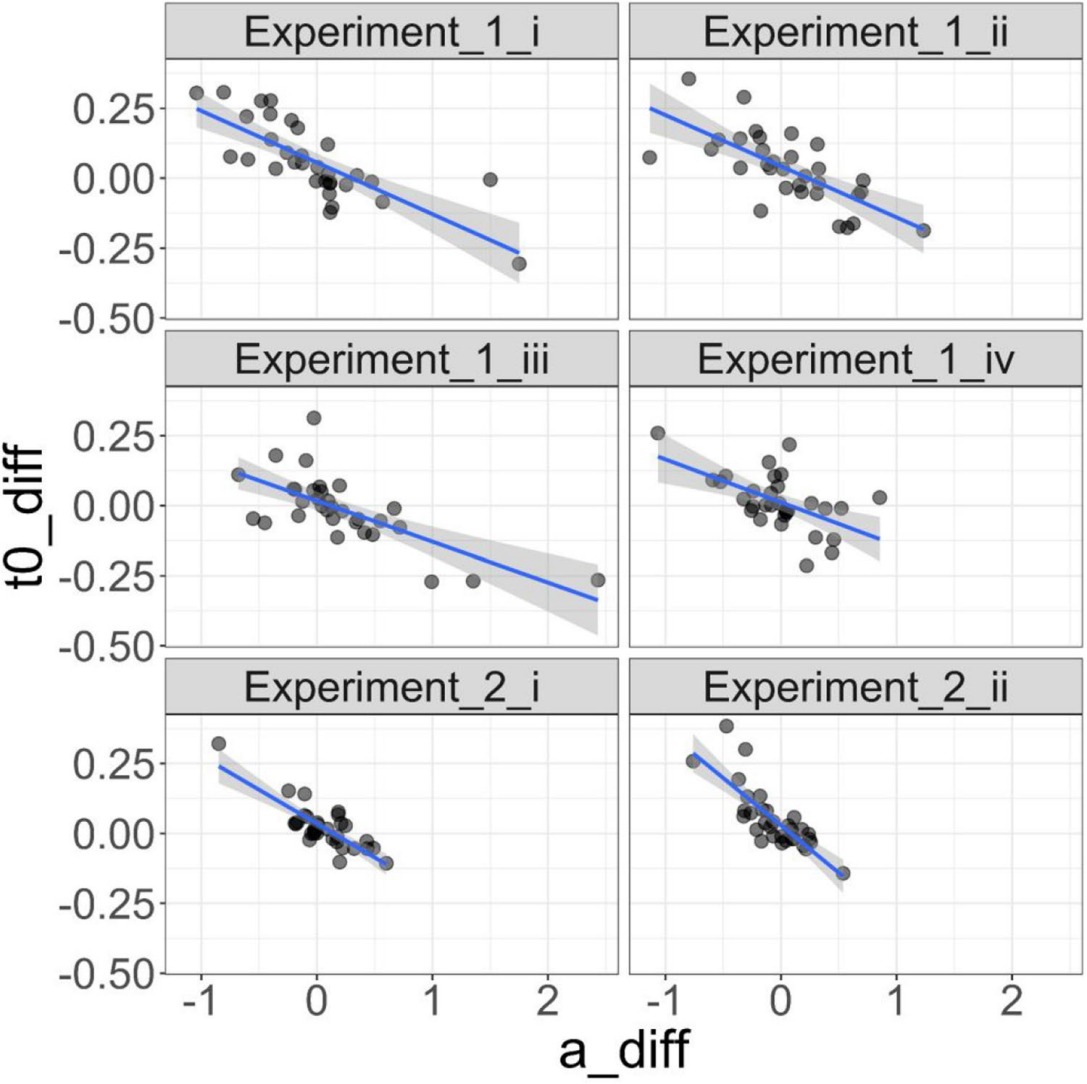
Plots of the correlation between *a*_difference and *t0*_difference from all conditions across Experiments 1 and 2 from the Schuch and Grange (2019). *Points* represent individual participant difference scores in diffusion model parameters and *lines* represent a linear model (with shading denoting 95% confidence intervals around the linear model predictions)

In Experiment 1 from Schuch and Grange (2019) with *N* = 32, and about 100 trials per condition and participant (mean = 102, SD = 10, min. = 53, max. = 121), the correlation between the *a-*difference and the *t0*-difference was *r* = – 0.767, *r* = – 0.706, *r* = – 0.701, and *r* = – 0.565, in conditions I, II, III, and IV, respectively (see Table 1). These correlations are visualised in Fig. 2. At the same time, the *a-*difference was correlated moderately positive with the *v-*difference (*r* = 0.398, r = 0.355, *r* = 0.543, *r* = 0.327, in conditions I, II, III, and IV); correlations between *t0*-difference and *v-*difference were weak or close to zero (*r* = – 0.063, *r* = 0.182, *r* = – 0.078, *r* = 0.298 in conditions I, II, III, and IV, respectively).

**Table 1:** Product-moment correlation coefficients between the fitted parameters from fast-dm-30 from Schuch and Grange (2019), Experiment 1. easy: CBA condition; hard: ABA condition; diff: hard-minus-easy difference in the corresponding parameter; conditions I-IV: different experimental conditions; see main text for details

	*a* (easy)	*a* (hard)	*v* (easy)	*v* (hard)	*t0* (easy)	*t0* (hard)	*a* diff	*v* diff	*t0* diff
*a* (easy)	—								
*a* (hard)	I: 0.725 II: 0.803 III: 0.723 IV: 0.843	—							
*v* (easy)	I: – 0.427 II: – 0.362 III: – 0.575 IV: – 0.711	I: – 0.551 II: – 0.501 III: – 0.531 IV: – 0.691	—						
*v* (hard)	I: – 0.603 II: – 0.580 III: – 0.652 IV: – 0.727	I: – 0.496 II: – 0.531 III: – 0.343 IV: – 0.616	I: 0.705 II: 0.666 III: 0.782 IV: 0.885	—					
*t0* (easy)	I: – 0.084 II: – 0.161 III: 0.050 IV: 0.154	I: 0.127 II: – 0.034 III: 0.357 IV: 0.250	I: – 0.187 II: – 0.137 III: – 0.116 IV: – 0.174	I: – 0.080 II: 0.178 III: – 0.138 IV: – 0.189	—				
*t0* (hard)	I: 0.039 II: – 0.118 III: 0.048 IV: 0.167	I: – 0.109 II: – 0.223 III: 0.111 IV: 0.134	I: – 0.174 II: – 0.149 III: – 0.130 IV: – 0.201	I: – 0.118 II: 0.204 III: – 0.182 IV: – 0.148	I: 0.803 II: 0.865 III: 0.857 IV: 0.910	—			
*a* diff	I: – 0.196 II: – 0.208 III: – 0.056 IV: – 0.106	I: 0.532 II: 0.416 III: 0.650 IV: 0.446	I: – 0.259 II: – 0.270 III: – 0.135 IV: – 0.093	I: 0.035 II: 0.015 III: 0.222 IV: 0.071	I: 0.285 II: 0.191 III: – 0.461 IV: 0.206	I: – 0.204 II: – 0.186 III: 0.107 IV: – 0.031	—		
*v* diff	I: 0.028 II: – 0.241 III: – 0.157 IV: 0.193	I: 0.304 II: – 0.008 III: 0.256 IV: 0.351	I: – 0.737 II: – 0.453 III: – 0.270 IV: – 0.522	I: – 0.041 II: 0.363 III: 0.389 IV: – 0.064	I: 0.187 II: 0.384 III: – 0.041 IV: 0.028	I: 0.134 II: 0.430 III: – 0.089 IV: 0.160	I: 0.398 II: 0.355 III: 0.543 IV: 0.327	—	
*t0* diff	I: 0.192 II: 0.048 III: – 0.011 IV: 0.015	I: – 0.373 II: – 0.385 III: – 0.494 IV: – 0.292	I: – 0.004 II: – 0.057 III: – 0.004 IV: – 0.043	I: – 0.072 II: 0.093 III: – 0.054 IV: 0.112	I: – 0.191 II: – 0.035 III: – 0.422 IV: – 0.308	I: 0.431 II: 0.471 III: 0.106 IV: 0.115	I: – 0.767 II: – 0.706 III: – 0.701 IV: – 0.565	I: – 0.063 II: 0.182 III: – 0.078 IV: 0.298	—

### Experiment 2 from Schuch and Grange (2019)

Experiment 2 from Schuch and Grange (2019) with *N* = 32 new participants was similar to Experiment 1, but involved a larger number of trials. In Experiment 2, a task-preparation interval of intermediate length was used in three out of four trials; only in every fourth trial, the task-preparation interval was long or short. When analysing the trials with intermediate task-preparation interval, a larger number of trials per condition was available (mean = 285, SD = 35, min. = 186, max. = 346). For these trials, there were only two experimental conditions (condition I: task sequence preceded by another ABA sequence; condition II: task sequence preceded by another CBA sequence). Again, there was a pronounced negative correlation between *a-*difference and *t0*-difference, which amounted to *r* = – 0.81 in both experimental conditions (see Table 2, again visualised in Fig. 2). At the same time, there were no strong correlations between *a-*difference and *v*-difference (*r* = 0.15 and *r* = 0.03 in conditions I and II, respectively) or between *t0*-difference and *v-*difference (*r* = 0.16 and *r* = 0.41 in conditions I and II).

**Table 2:** Product-moment correlation coefficients between the fitted parameters from fast-dm-30 from Schuch and Grange (2019), Experiment 2, trials with intermediate CSI. easy: CBA condition; hard: ABA condition; diff: hard-minus-easy difference in the corresponding parameter; conditions I-II: different experimental conditions; see main text for details

	*a* (easy)	*a* (hard)	*v* (easy)	*v* (hard)	*t0* (easy)	*t0* (hard)	*a* diff	*v* diff	*t0* diff
*a* (easy)	—								
*a* (hard)	I: 0.846 II: 0.846	—							
*v* (easy)	I: – 0.530 II: – 0.410	I: – 0.587 II: 0.372	—						
*v* (hard)	I: – 0.446 II: – 0.261	I: – 0.501 II: – 0.200	I: 0.912 II: 0.835	—					
*t0* (easy)	I: – 0.098 II: – 0.223	I: – 0.122 II: – 0.310	I: 0.044 II: 0.055	I: – 0.018 II: – 0.012	—				
*t0* (hard)	I: 0.007 II: – 0.117	I: – 0.233 II: – 0.185	I: – 0.008 II: – 0.071	I: – 0.014 II: 0.005	I: 0.883 II: 0.836	—			
*a* diff	I: 0.020 II: – 0.429	I: 0.551 II: 0.119	I: – 0.271 II: 0.132	I: – 0.240 II: 0.147	I: – 0.075 II: – 0.108	I: – 0.447 II: 0.531	—		
*v* diff	I: 0.348 II: 0.262	I: 0.372 II: 0.303	I: – 0.510 II: – 0.296	I: – 0.112 II: 0.278	I: – 0.145 II: – 0.116	I: – 0.049 II: 0.133	I: 0.153 II: 0.026	—	
*t0* diff	I: 0.193 II: 0.534	I: – 0.273 II: 0.110	I: – 0.063 II: – 0.209	I: 0.003 II: 0.026	I: 0.060 II: 0.079	I: 0.522 II: 0.613	I: – 0.813 II: – 0.808	I: 0.159 II: 0.409	—

We also observed the negative correlation between *a-*difference and *t0*-difference in another empirical data set (task-switching paradigm; unpublished data); there we checked whether this correlation depends on particularities of the diffusion-model settings. We still observed the correlation when using the maximum-likelihood (ML) instead of the KS criterion for the fitting procedure, and when allowing *st0* to vary across conditions versus fixing it across experimental conditions (both changes were inspired by the diffusion-modelling procedure reported in Janczyk & Lerche, 2019). We observed the spurious correlation in all four combinations of fitting criterion (ML versus KS) and *st0* setting (variable versus fixed across conditions); moreover, when using the EZ-diffusion model (Wagenmakers et al., 2007) instead of *fast-dm*, we again obtained similar results.

To summarize, we consistently observed a pronounced negative correlation between *a-*difference and *t0*-difference when applying a simple drift-diffusion model to empirical task-switching data. The correlation occurred in different experimental conditions, and with different diffusion-model settings.

## Reanalysis of Dutilh et al. (2019)

To check for the generality of this finding, in a next step we reanalysed an empirical data set collected independently from our own research groups. We selected the data set of Dutilh et al. (2019), which was used in a “many-analysts” examination of the variability of model-based inferences of two-choice RT data. Dutilh et al. created 14 data sets; each data set comprised two conditions, and in most data sets some aspect of participants’ behaviour was manipulated to selectively induce changes in model parameters across two conditions (“Condition A” and “Condition B”). The primary task in all experiments was a random dot motion task where a cloud of dots was presented to participants; a proportion of the dots moved in a consistent direction, and the remainder moved in a random direction. The task required participants to judge the direction of the coherently moving dots.

Of the 14 data sets, we reanalysed three; these were selected as either no DDM parameter was expected to change across conditions (i.e., data set from Experiment 1), or only one parameter was expected to change across conditions (i.e., data sets from Experiments 2 & 3). In the data set from Experiment 1, there were no experimental differences between the two conditions, and therefore there should be no differences in DDM parameters across conditions. Therefore, the labelling of “Condition A” and “Condition B” is somewhat arbitrary in this Experiment, but it does allow us to explore whether the spurious correlation is present in a data set with no condition-differences in model parameters. The data comprising Experiment 2 was obtained via a manipulation of task difficulty between Conditions A and B; specifically, the proportion of coherently moving dots in the cloud presented on each trial was 20% in Condition A, and 10% in Condition B; Condition B can thus be considered a “hard” condition, and Condition A a (relatively) “easy” condition. The data comprising Experiment 3 was obtained via a manipulation of response caution between Conditions A and B; specifically, in Condition A participants were instructed to respond with an emphasis on *speed*, and to respond with an emphasis on *accuracy* in Condition B. This speed–accuracy manipulation influences the response boundary parameter typically in drift-diffusion modelling (see e.g., Heitz, 2014).

The data for each experiment—and for each condition within each experiment—was extracted as described in Dutilh et al. (2019) and fit with *fast-dm-30* using maximum likelihood criterion with *v*, *a*, and *t0* free to vary across conditions A and B. The model was fit to each experiment separately. The correlation coefficients among all parameters are shown in Table 3.

**Table 3:** Product-moment correlation coefficients between the fitted parameters from fast-dm-30 from Dutilh et al. (2019) Experiments 1–3 (conditions A and B). diff = difference score for model parameters calculated as condition B minus condition A. See main text for details

	*a* (A)	*a* (B)	*v* (A)	*v* (B)	*t0* (A)	*t0* (B)	*a* diff	*v* diff	*t0* diff
*a* (A)	—								
*a* (B)	1: 0.327 2: 0.269 3: – 0.177	—							
*v* (A)	1: 0.090 2: – 0.125 3: 0.172	I: – 0.247 2: 0.286 3: – 0.072	—						
*v* (B)	1: 0.021 2: – 0.167 3: 0.332	1: – 0.161 2: 0.231 3: – 0.267	1: 0.907 2: 0.966 3: 0.689	—					
*t0* (A)	1: – 0.483 2: – 0.406 3: – 0.324	1: – 0.091 2: – 0.008 3: 0.089	1: 0.549 2: 0.673 3: 0.422	1: 0.568 2: 0.708 3: – 0.050	—				
*t0* (B)	1: 0.085 2: 0.100 3: – 0.362	1: – 0.358 2: – 0.374 3: 0.096	1: 0.712 2: 0.311 3: 0.005	1: 0.673 2: 0.386 3: – 0.182	1: 0.702 2: 0.675 3: 0.572	—			
*a* diff	1: – 0.749 2: – 0.642 3: – 0.510	1: 0.381 2: 0.566 3: 0.937	1: – 0.261 2: 0.335 3: – 0.124	1: – 0.133 2: 0.327 3: – 0.351	1: 0.408 2: 0.341 3: 0.193	1: – 0.335 2: – 0.383 3: 0.212	—		
*v* diff	1: – 0.161 2: 0.065 3: 0.130	1: 0.202 2: – 0.326 3: – 0.190	1: – 0.231 2: – 0.952 3: – 0.606	1: 0.201 2: – 0.840 3: 0.160	1: 0.035 2: – 0.572 3: – 0.629	1: – 0.100 2: 0.197 3: – 0.206	1: 0.299 2: – 0.315 3: – 0.216	—	
*t0* diff	1: 0.747 2: 0.616 3: – 0.101	1: – 0.332 2: – 0.467 3: 0.023	1: 0.174 2: – 0.410 3: – 0.408	1: 0.100 2: – 0.358 3: – 0.161	1: – 0.435 2: – 0.338 3: – 0.319	1: 0.335 2: 0.466 3: 0.595	1: – 0.964 2: – 0.899 3: 0.056	1: – 0.173 2: 0.435 3: 0.379	—

The negative correlation between *a*-difference and *t0*-difference was very large in Experiment 1 (*r* = – 0.964) and Experiment 2 (*r* = – 0.899), but was absent in Experiment 3 (*r* = 0.056). At the same time, the *a-*difference was correlated moderately positively with the *v-*difference in Experiment 1 (*r* = 0.299), but this correlation was moderate and *negative* in Experiments 2 (*r* = – 0.315) and 3 (*r* = – 0.216); the correlation between *t0*-difference and *v-*difference was small and negative in Experiment 1 (*r* = – 0.173), but was moderate and positive in Experiments 2 (*r* = 0.435) and 3 (*r* = 0.379).

To summarise the reanalysis of Dutilh et al. (2019), we found the spurious correlation between *a*-difference and *t0*-difference in two of the three data sets. Surprisingly, we did not find the correlation in Experiment 3. In that experiment, response caution was manipulated via speed–accuracy emphasis instructions; such instructions have been shown to selectively influence estimates of boundary separation in drift-diffusion model fitting (e.g., Forstmann et al., 2011; Heitz, 2014; but see Rae et al., 2014). This raises an interesting possibility that the spurious correlation observed between *a*-difference and *t0*-difference only occurs when the experimental manipulations do not affect the boundary separation parameter. We explore this question—among others—in a set of simulations studies.

## Simulation studies

In this section, we conducted a series of simulations to explore the correlation between *a*-difference and *t0*-difference. We were primarily interested in understanding whether the observed correlation was peculiar to the empirical data sets investigated above or whether it was a general result that might be found in other situations. Simulations are important to explore such questions as we are able to generate synthetic data where the values for the true data-generating parameters are known, which is not possible with real data.

### Simulation 1: No difference in parameters

In Simulation 1, we wanted to explore whether we would observe the correlation between *a*-difference and *t0*-difference in a data set where no true difference exists in any of the main DDM parameters between two experimental conditions. This provides a strong test of whether the observed correlation is an artefact of real behavioural data or whether it is an emergent and general property of DDM model fits: If we observe a correlation between *a*-difference and *t0*-difference in the absence of a true difference in parameter values between conditions then the correlation must be a general property of DDM fitting. This simulation is thus similar in design to Experiment 1 from Dutilh et al. (2019), where there was no experimental manipulation of participant behaviour between the two experimental conditions.

We conducted a simulation where data were generated from 1000 artificial participants in two experimental conditions; we refer to these as an “easy” condition and a “hard” condition throughout, even though in this particular simulation no differences in parameters existed. For each participant, 1000 trials were simulated in each condition; such large trial numbers have been shown to lead to excellent parameter recovery (see Lerche et al., 2017) and as such noise in the parameter estimation routine is minimised. In Simulation 1, the diffusion parameters used to generate the data were randomly selected for each participant, but importantly the exact same parameters were used for each participant to generate data in both the easy and the hard condition. Specifically, parameters for each participant in the easy condition were sampled from a uniform distribution with the following minimum and maximum values: *v* [0.0–4.0], *a* [0.5–2.0], and *t0* [0.2–0.5] (see Lerche et al., 2017, for similar values); the exact same parameters for the easy condition were then used for the hard condition. Throughout all simulations reported in this paper, we fixed the starting point of the diffusion process equidistant between response boundaries (i.e., parameter *zr* was set to 0.5), and all variability parameters were set to zero. Data were simulated using the *fast-dm-30* software (Voss et al., 2015) using the *construct-samples* routine with precision set to 3^1^. The simulated behavioural data showed similar response times in the easy condition (M = 0.625 s, SD = 0.206 s) and the hard condition (M = 0.626 s, SD = 0.207 s), and the accuracy was identical in both conditions (M = 84.6%, SD = 14.4%).

The diffusion model was then fit to the simulated behavioural data. During fitting, only the three main parameters—*v*, *a*, and *t0*—were allowed to freely vary across conditions. The starting point parameter *zr* was fixed at 0.5, and all variability parameters were fixed at zero^2^. Maximum likelihood was used as the optimisation criterion throughout all simulations. Recovery of the generating parameter values used to generate simulated data was excellent in the fitting routine. The correlation between the parameter values used to generate simulated data and the recovered best-fitting parameter values were all above *r* = 0.995 (see Appendix A).

The fit routine returned the set of best-fitting parameters per participant, namely *v* (easy), *v* (hard), *a* (easy), *a* (hard), *t0* (easy), and *t0* (hard). We then calculated difference scores on each parameter, calculated as the estimate for the easy condition subtracted from the estimate for the hard condition, namely *v-*difference, *a-*difference, and *t0-*difference. We then computed the product-moment correlation coefficient matrix across all of the parameters. Due to the large participant numbers in the simulation, we did not calculate statistical significance. Instead, we interpret the correlation coefficient as an effect size, and use *r* = |0.1| to denote a small effect, *r* = |0.3| to denote a medium effect, and *r* >= |0.5| to denote a large effect. The correlation matrix is shown in Table 4.

**Table 4:** Product-moment correlation coefficients between the fitted parameters from the *fast-dm-30* fitting routine in Simulation 1. Diff = difference scores on parameters (hard minus easy)

	*a* (easy)	*a* (hard)	*v* (easy)	*v* (hard)	*t0* (easy)	*t0* (hard)	*a* diff	*v* diff	*t0* diff
*a* (easy)	—								
*a* (hard)	0.991	—							
*v* (easy)	0.043	0.039	—						
*v* (hard)	0.009	0.042	0.994	—					
*t0* (easy)	– 0.045	– 0.041	– 0.001	– 0.001	—				
*t0* (hard)	– 0.049	– 0.052	0.005	0.003	0.998	—			
*a* diff	– 0.020	0.117	– 0.026	0.025	0.027	– 0.022	—		
*v* diff	– 0.038	0.027	– 0.072	0.036	– 0.002	– 0.017	0.473	—	
*t0* diff	– 0.056	– 0.158	0.080	0.057	– 0.023	0.043	– 0.743	– 0.215	—

As can be seen, we found a large negative correlation (*r* = – .743) between the difference score in boundary separation (*a-*difference) and the difference score in non-decision time (*t0-*difference), replicating the finding in the Schuch and Grange (2019) data set, as well as Experiments 1 and 2 from the reanalysis of Dutilh et al. (2019). This correlation was present despite there being no correlation between the boundary separation parameter and the non-decision time parameter for either the easy or the hard conditions. Aside from the expected large correlations between matching parameters across conditions (e.g., *v*[easy] and *v*[hard]), no other correlations were above medium in effect size. Within the difference scores, we observed a medium positive correlation between *a*-difference and *v*-difference which we also observed in the reanalysis of Experiment 1 of Schuch and Grange (2019), and in Experiment 1 of the reanalysis of Dutilh et al. (2019). In addition, there was a small negative correlation between *v*-difference and *t0*-difference, which we did not observe consistently in the real data sets.

To check that the observed correlations were not due to the model estimation programme used, we fitted the EZ-diffusion model (Wagenmakers et al., 2007) to the behavioural data generated by the simulation^3^. The correlation matrix for this fitting routine is shown in Table 5, and shows qualitatively similar results, and in particular the correlation between *a*-difference and *t0*-difference remained large and negative. Note, though, that the correlation between *a*-difference and *v*-difference—whilst still positive—reduced in size to a small effect. The small negative correlation between *v*-difference and *t0*-difference was absent in the EZ model fit.

**Table 5:** Product-moment correlation coefficients between the fitted parameters from the EZ-diffusion fitting routine in Simulation 1. Diff = difference scores on parameters (hard minus easy)

	*a* (easy)	*a* (hard)	*v* (easy)	*v* (hard)	*t0* (easy)	*t0* (hard)	*a* diff	*v* diff	*t0* diff
*a* (easy)	—								
*a* (hard)	0.978	—							
*v* (easy)	0.033	0.040	—						
*v* (hard)	0.033	0.047	0.993	—					
*t0* (easy)	– 0.062	– 0.039	0.008	0.005	—				
*t0* (hard)	– 0.057	– 0.067	– 0.003	– 0.004	0.974	—			
*a* diff	– 0.015	0.192	0.037	0.070	0.104	– 0.053	—		
*v* diff	– 0.003	0.053	– 0.070	0.050	– 0.024	– 0.012	0.273	—	
*t0* diff	0.024	– 0.118	– 0.046	– 0.039	– 0.174	0.055	– 0.683	0.053	—

### Simulation 2: Introducing differences in each main parameter

Although Simulation 1 provides a strong test of the correlation between *a*-difference and *t0*-difference due to no true parameter difference between conditions, it does present a somewhat unrealistic representation of the type of real data analysts are likely to use the diffusion model for, where some experimental manipulation is known to lead to differences between conditions. In Simulation 2 we therefore explored the impact of a true condition difference on the observed negative correlation between *a*-difference and *t0*-difference. We conducted a series of simulations wherein we systematically generated artificial data where just one parameter changed between easy and hard conditions, whilst the other parameters remained fixed. We repeated the simulations to generate data that exhibited a small (Cohen’s *d* = 0.3), medium (*d* = 0.5), and large (*d* = 0.8) effect size. This allows us to explore the impact of selectively changing just one parameter between two conditions on the correlation between *a*-difference and *t0*-difference. After this, we then explored the effect of changing all parameters between conditions.

#### Changing one main DDM parameter between conditions

The values for the manipulated parameter for simulated participants were sampled from a multivariate normal distribution (with the constraint that all parameters should be positive values) with means and standard deviations that allowed the various effect sizes of interest. For example, when the drift rate was manipulated to have a small effect size across conditions, the parameter values for drift rate were sampled from a multivariate normal distribution with means M(easy) = 2.3, M(difficult) = 2.0, and standard deviations SD(easy) = 1, SD(hard) = 1, constrained to have a correlation *r* = 0.5 between the two parameters at the population level. The other parameters were selected as in Simulation 1 by sampling parameters for the easy condition from a uniform distribution and using the exact same parameters in the hard condition (see Table 6 for full parameter-generating details). These parameter values were then used to simulate data using the *construct-samples* routine in *fast-dm-30*.

**Table 6:** Parameter sampling values used to generate simulated data in Simulation 2. Each row represents a separate sub-simulation, wherein one key parameter was manipulated to have either a small, medium, or large effect across easy and hard conditions

Sub-simulation	*v* (easy)	*v* (hard)	*a* (easy)	*a* (hard)	*t0* (easy)	*t0* (hard)
*v* (small effect)	μ = 2.3, σ = 1	μ = 2.0, σ = 1	unif[0.5–2.0]	= *a* (easy)	unif[0.2–0.5]	= *t0* (easy)
*v* (medium effect)	μ = 2.5, σ = 1	μ = 2.0, σ = 1	unif[0.5–2.0]	= *a* (easy)	unif[0.2–0.5]	= *t0* (easy)
*v* (large effect)	μ = 2.8, σ = 1	μ = 2.0, σ = 1	unif[0.5–2.0]	= *a* (easy)	unif[0.2–0.5]	= *t0* (easy)
*a* (small effect)	unif[0.0–4.0]	= *v* (easy)	μ = 1.25, σ = 0.4	μ = 1.37, σ = 0.4	unif[0.2–0.5]	= *t0* (easy)
*a* (medium effect)	unif[0.0–4.0]	= *v* (easy)	μ = 1.25, σ = 0.4	μ = 1.45, σ = 0.4	unif[0.2–0.5]	= *t0* (easy)
*a* (large effect)	unif[0.0–4.0]	= *v* (easy)	μ = 1.25, σ = 0.4	μ = 1.57, σ = 0.4	unif[0.2–0.5]	= *t0* (easy)
*t0* (small effect)	unif[0.0–4.0]	= *v* (easy)	unif[0.5–2.0]	= *a* (easy)	μ = 0.350, σ = 0.1	μ = 0.385, σ = 0.1
*t0* (medium effect)	unif[0.0–4.0]	= *v* (easy)	unif[0.5–2.0]	= *a* (easy)	μ = 0.350, σ = 0.1	μ = 0.400, σ = 0.1
*t0* (large effect)	unif[0.0–4.0]	= *v* (easy)	unif[0.5–2.0]	= *a* (easy)	μ = 0.350, σ = 0.1	μ = 0.430, σ = 0.1

Where a parameter is manipulated between conditions, μ and σ provide the mean and standard deviation (respectively) for each condition used to draw samples from a multivariate normal distribution with correlation *ρ* = 0.5 between the manipulated parameters. = signifies identical parameters were used to the named column (e.g., = *t0* (easy) means the parameters were identical to those in the *t0* (easy) column. μ = mean of the condition. σ = standard deviation of the condition. Unif refers to a uniform distribution with the range of possible values shown in square brackets

We generated parameter values for 1000 artificial participants in each factorial combination of manipulated parameter and effect size. For each combination of manipulated parameter and effect size, 1000 trials were simulated per condition per participant. The diffusion model was then fitted to the simulated data in the same way as in Simulation 1.

**Fig. 3:**
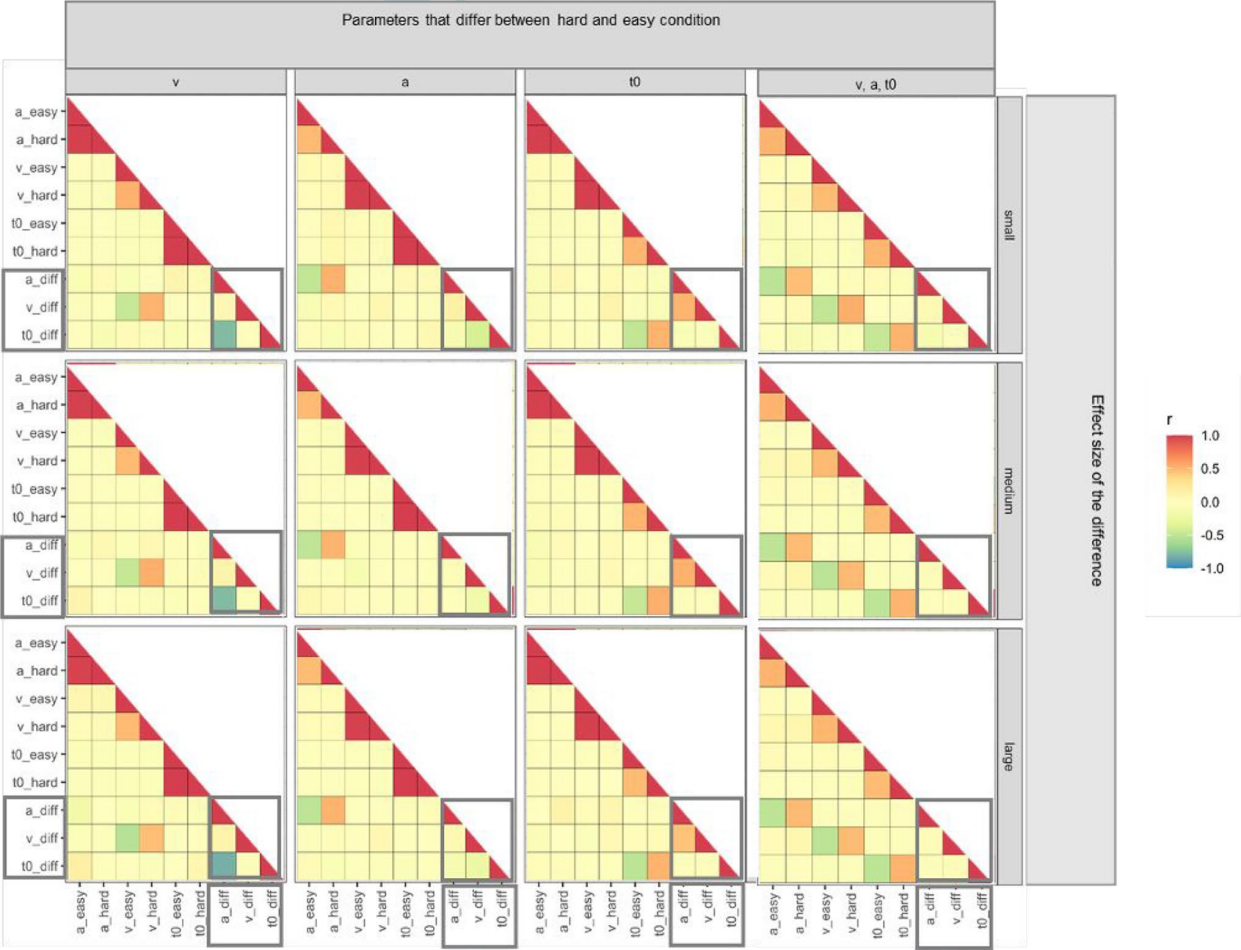
Heatmaps of the product-moment correlation matrices from Simulation 2 where one DDM parameter was changed across conditions, and Simulation 3 where all three parameters were changed across conditions. *Columns* represent which parameters were manipulated to be different between simulated conditions, and *rows* represent the effect size of the manipulated difference in those parameters. Diff = difference score. Correlations between difference scores are highlighted by *grey rectangles*

The results of the simulations are visualised in Fig. 3, which shows heatmaps of the correlation matrices for each of the nine combinations of which parameter was manipulated, and to what effect size. The correlation matrices are presented numerically in Appendix B, together with the numerical correlation matrices resulting from fitting the simulated data with the EZ-diffusion model.

The results showed quite strikingly that the effects of which parameter was manipulated produced remarkably consistent outcomes across all effect sizes (that is, the pattern of correlations in each column is consistent across each effect size magnitude).

**Fig. 4:**
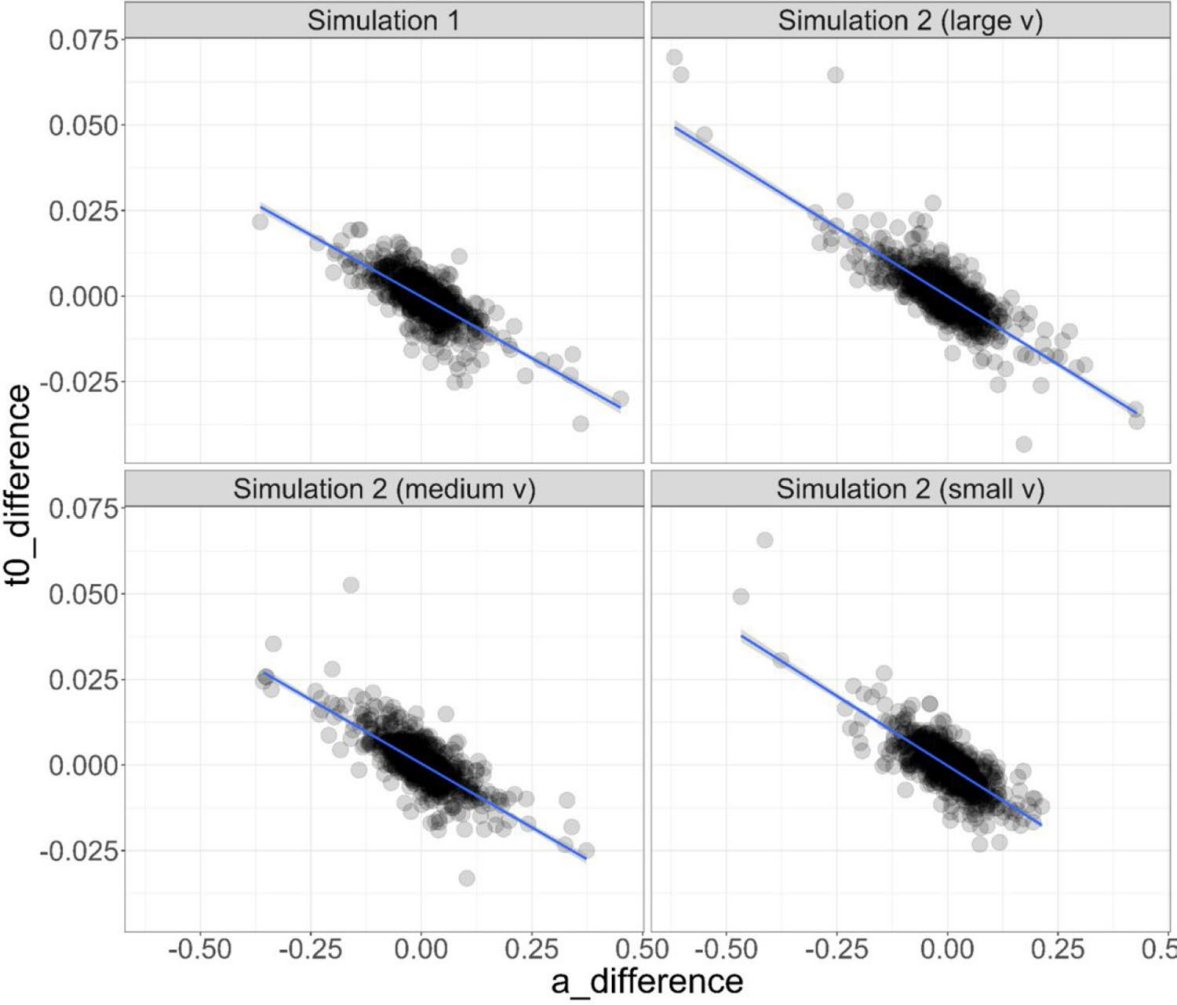
Plots of the correlation between *a*-difference and *t0*-difference in Simulation 1, and Simulation 2 (with drift rate, *v*, manipulated to have a small, medium, and large difference across conditions). *Points* represent simulated participants’ difference scores in diffusion model parameters, and the *lines* represent a linear model (with shading—barely visible—denoting 95% confidence intervals around the linear model predictions)

The negative correlation between *a*-difference and *t0*-difference was only present when drift rate was varied across conditions (column 1 of Fig. 3), but not when boundary separation or non-decision time, or all three parameters, were varied across conditions (columns 2–4 of Fig. 3). The negative correlation between *a*-difference and *t0*-difference was large across each manipulated effect size of the drift rate effect (*r* = – 0.774 when *d* was small, *r* = – 0.769 when *d* was medium, and *r* = – .819 when *d* was large). These correlations are visualised in Fig. 4.

These correlations were not driven by outlier scores. We assessed the impact of outliers by standardising the data and removing any simulated participant with standardised scores lower than – 2.5 or higher than 2.5. The correlations remained negative: *r*s = – 0.661, – 0.693, – 0.688, – 0.705 for Simulation 1, Simulation 2 (small v), Simulation 2 (medium v), and Simulation 2 (large v) respectively.

When t0 was manipulated, we found a moderate positive correlation between *a*-difference and *v*-difference, which ranged from *r* = 0.455–0.521. These correlations were present in Experiment 1 of Schuch and Grange (2019; ranging from *r* = 0.33 to 0.54) and in Experiment 1 of Dutilh et al. (2019; *r* = 0.30); this correlation was absent from the reanalysis of Experiment 2 of Schuch and Grange, but was medium and negative in Experiments 2 and 3 of Dutilh et al. (*r*s = – 0.32 and – 0.22, respectively). Similar to the correlation between *a*-difference and *t0*-difference, these correlations were not driven by outlier scores. When we standardised the scores and removed any simulated participant with standardised scores lower than – 2.5 or higher than 2.5, the correlations were *r*s = 0.350, 0.389, 0.355, 0.328 for Simulation 1, Simulation 2 (small v), Simulation 2 (medium v), and Simulation 2 (large v), respectively.

No other correlations were consistently found between difference parameters. Note that although there was a medium negative correlation between *v*-difference and *t0*-difference when boundary separation was manipulated to a small effect size, this did not replicate at other effect-size manipulations, nor was it present in the EZ-diffusion fitting.

The only other correlations present are to be expected from the design of the simulations. Namely, when a parameter was not manipulated between conditions, a large positive correlation was found between its fitted estimate in easy and hard conditions. When a parameter was manipulated, the fitted estimate for the easy condition was moderately positively correlated with the hard condition (because the data were generated to have an *r* = .5 correlation). In addition, when a parameter was manipulated, the estimates of that parameter for the easy and the hard condition were correlated with the magnitude of the difference score in that parameter (negatively for the easy-condition parameter, and positively for the hard-condition parameter). This latter pattern was found for the drift rate parameter in the reanalysis of Schuch and Grange (2019): *v*-difference was consistently negatively correlated with the *v* estimate in the easy conditions (ranging from *r* = – 0.27 to *r* = – 0.74), and in some conditions positively correlated with the *v* estimate in the hard condition (range *r* = – 0.11 to *r* = 0.39). In the reanalysis of Dutilh et al. (2019), *v*-difference was negatively correlated with the *v*-estimate in Condition A (notionally the “easy” condition; range *r* = – 0.23 to – 0.95), but was only positively correlated with the *v*-estimate in Condition B in Experiments 1 and 3 (*r*s = 0.20 and 0.16, respectively); in Experiment 2 (where drift rate was manipulated behaviourally), the correlation was large and negative (*r* = – 0.84).

#### Changing all main DDM parameters between conditions

In the next part of the simulation, we simulated data where all three main parameters changed between conditions, again manipulating the size of the changes to be small, medium, and large. The parameters were generated as presented in Table 7, except all three parameters changed across conditions. Again, 1000 participants were simulated with 1000 trials per condition. The results of the simulation are shown in Fig. 3, most-right column (numerical correlation matrices and EZ-diffusion results are in Appendix B). The results showed only the expected correlations dictated by the simulation design; the negative correlation between *a*-difference and *t0*-difference was not found.

**Table 7:** Product-moment correlation coefficients between the fitted parameters from the rtdists fitting routine in Simulation 3. Diff = difference scores on parameters (hard minus easy)

	*a* (easy)	*a* (hard)	*v* (easy)	*v* (hard)	*t0* (easy)	*t0* (hard)	*a* diff	*v* diff	*t0* diff
*a* (easy)	—								
*a* (hard)	0.991	—							
*v* (easy)	0.045	0.035	—						
*v* (hard)	0.050	0.048	0.994	—					
*t0* (easy)	– 0.051	– 0.048	– 0.006	– 0.004	—				
*t0* (hard)	– 0.044	– 0.048	– 0.002	– 0.003	0.998	—			
*a* diff	– 0.126	0.011	– 0.076	– 0.019	0.024	– 0.024	—		
*v* diff	0.043	0.115	– 0.099	0.013	0.011	– 0.004	0.513	—	
*t0* diff	0.098	– 0.004	0.052	0.026	– 0.009	0.056	– 0.741	– 0.240	—

## Discussion

Taken together, the results of Simulation 1 and Simulation 2 show that the negative correlation between *a*-difference and *t0*-difference found in the reanalysis of Schuch and Grange (2019) and in Experiments 1 and 2 from Dutilh et al. (2019) appears when there is either no true difference in parameter values across conditions (e.g., Experiment 1 in Dutilh et al., 2019), or the true difference is localised to the drift rate parameter (e.g., Experiments 1 & 2 from Schuch & Grange, 2019, and Experiment 2 from Dutilh et al., 2019). This conclusion is congruent with us not finding the correlation in the reanalysis of Experiment 3 from Dutilh et al. (2019), where the experiment’s speed–accuracy trade-off likely influenced the boundary separation parameter between conditions. In other words, the spurious correlation between *a*-difference and *t0*-difference emerges whenever there is no true difference in the *a* and *t0* parameters across conditions, irrespective of whether there is a true condition difference in drift rate or not.

### Simulation 3: Using *rtdists* package

In Simulations 1 and 2, we simulated and fitted the diffusion model using the *fast-dm-30* software provided by Voss et al. (2015). We wanted to assure ourselves that the observed negative correlation between *a*-difference and *t0*-difference was not due to the use of this software. Therefore, in Simulation 3 we utilised different simulation and fitting software. Specifically, we used the R package *rtdists* (Singmann et al., 2020)^4^ and repeated Simulation 1, where data are simulated with no true difference in parameters across conditions (again using the parameter generation criteria in Table 6). The results of the simulation are shown in the correlation matrix in Table 7.

Replicating Simulation 1, we found a large negative correlation between *a*-difference and *t0*-difference. Similar to Simulation 1, the positive correlation between *a*-difference and *v*-difference—this time with a large effect size—re-emerged, as did the small negative correlation between *v*-difference and *t0*-difference.

The finding of a large negative correlation between *a*-difference and *t0*-difference appears robust across simulation and fitting procedures. As also found in our behavioural data, we found in Simulation 1 a small positive correlation between *a*-difference and *v*-difference; although this was not replicated in the EZ-diffusion fit of the Simulation 1 dataset, the correlation has replicated using the *rtdists* package here in Simulation 3. In addition, Simulation 3 replicated the small negative correlation between *v*-difference and *t0*-difference, again generally consistent with our behavioural data reanalysis. Taken together, the findings of Simulation 3 broadly support the findings of Simulation 1: When no true differences exist in diffusion model parameters between experimental conditions, spurious correlations appear in all of the difference scores of the parameter values.

### Simulation 4: Linear ballistic accumulator model

In Simulation 3, we utilised a different simulation and model fitting environment to ensure our findings generalise across different implementations of the drift-diffusion model. In Simulation 4, we addressed whether the findings generalise to different theoretical accounts of evidence accumulation during rapid decision making. In particular, we simulated data from the Linear Ballistic Accumulator (LBA) model (Brown & Heathcote, 2008), and fitted the generated data with the LBA model.

**Fig 5:**
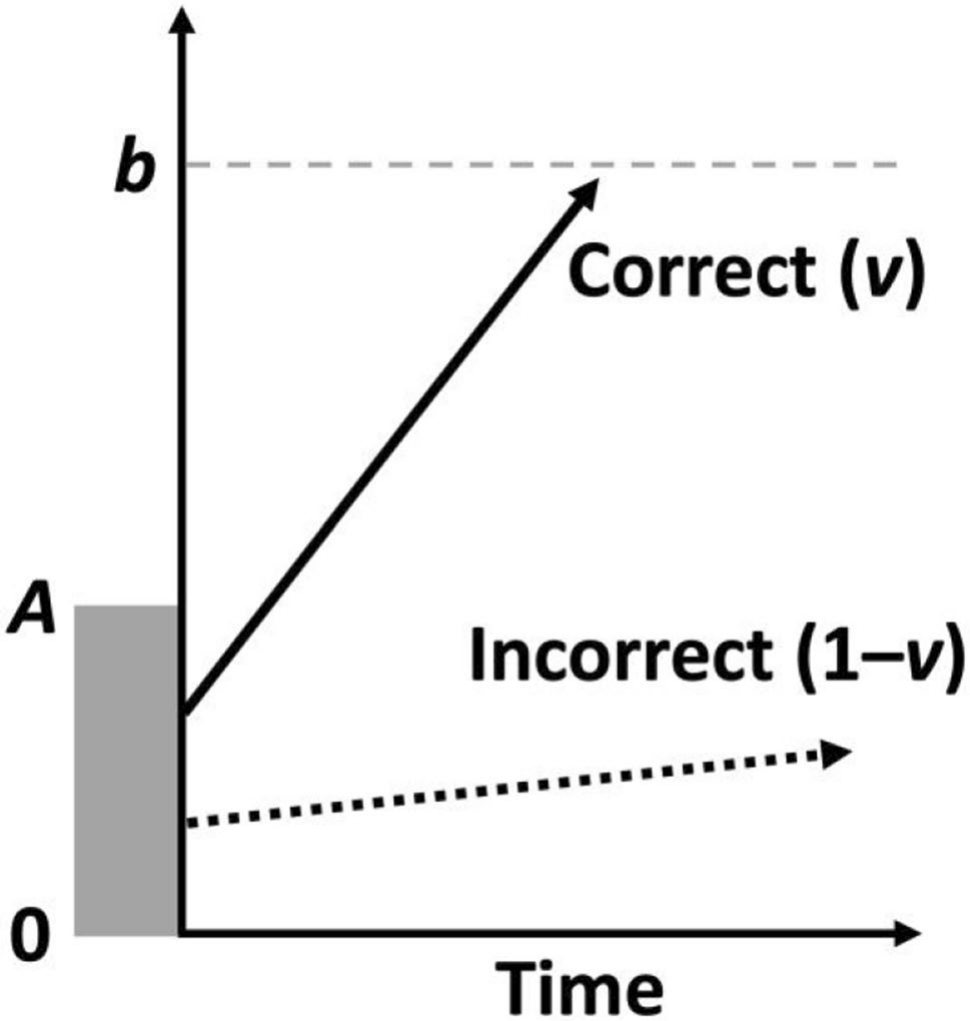
Schematic representation of trial processing in the Linear Ballistic Accumulator Model. Fig. available at https://www.flickr.com/photos/150716232@N04/51602517573 under CC license https://creativecommons.org/licenses/by/2.0/

The LBA model is similar to the DDM in that it decomposes response times into a decisional and non-decisional component. The decisional component of the LBA assumes—like the DDM—that responses are determined by an evidence-accumulation process (see Fig. 5 for a schematic overview of the LBA model). However, the LBA assumes that the evidence-accumulation process is linear, with the rate of evidence accumulation determined by the drift rate. The drift rate varies on each trial (modelled as a draw from a random distribution with mean *v* and standard deviation *s*), which models trial-wise variability in response time. The LBA assumes separate accumulators for each response option (cf., the DDM which assumes a single accumulator can hit one of two response boundaries; see Fig. 1), which in our simulations we generalise to include one accumulator for the “correct” response (with mean drift rate *v*) and one accumulator for the incorrect response (with mean drift rate 1–*v*). Each accumulator begins each trial with a certain amount of residual evidence, represented by a random starting point of the drift rate between 0–*A*, where *A* is a parameter that represents the height of the starting point boundary. A response is selected when an accumulator reaches the threshold (the height of which is represented by parameter *b*, which is constrained to be larger than *A*); RT is determined by the time taken for the first accumulator to reach the threshold, and accuracy is determined by whether the accumulator representing the correct response option was the first to reach the boundary or not. Response caution—equivalent to the boundary separation parameter *a* in the DDM—is given by *b* – (*A*/2). As with the standard diffusion model, the LBA model also has a parameter reflecting non-decisional components of performance (i.e., *t0*).

We simulated 1000 participants from the LBA model, with 1000 trials per condition. As in Simulation 1, the parameters were identical for each participant in each condition. All LBA parameters were drawn from uniform distributions with the following minimum and maximum values: mean correct *v* [1.0–4.0], *A* [0.5–2.0], *b* [A + 0.001–2.0], and *t0* [0.2–0.5]. The LBA model was then fitted to the generated data using a gradient descent method (as implemented in R’s *nlminb* function) to find the best set of LBA parameters for each participant and each condition that minimises the negative log-likelihood of the data. Starting parameters for the search routine were generated for each participant and each condition using the following heuristics (see Donkin et al., 2011): *t0* was set at 90% of the fastest response in the data; mean correct drift rate *v* was set to 0.5 + Φi^-1^(p), where p is the probability of a correct response, and Φ is the normal cumulative distribution function with mean equal to zero and standard deviation 0.3; *A* was set as twice the value of the inter-quartile range of all response times; and *b* was set to *A* × 1.25.

After the fit routine had found the best-fitting parameters for each participant and each condition, we calculated response caution for each participant and condition (calculated as *b*–[*A*/2]) and calculated difference scores for all main parameters (the easy condition parameter value subtracted from the hard condition parameter value). The correlation matrix is shown in Table 8.

**Table 8:** Product-moment correlation coefficients between the fitted parameters from the LBA simulation and fitting routine in Simulation 4. Note that caution was calculated as *b* - (*A*/2). Diff = difference scores on parameters (hard minus easy)

	Caution (easy)	Caution (hard)	*v* (easy)	*v* (hard)	*t0* (easy)	*t0* (hard)	Caution diff	*v* diff	*t0* diff
caution (easy)	—								
caution (hard)	0.925	—							
*v* (easy)	0.082	0.070	—						
*v* (hard)	0.060	0.103	0.979	—					
*t0* (easy)	–0.147	–0.039	–0.066	–0.052	—				
*t0* (hard)	–0.063	–0.165	–0.084	–0.095	0.765	—			
caution diff	–0.156	0.231	–0.018	0.112	0.277	–0.267	—		
*v* diff	–0.105	0.141	–0.080	0.125	0.068	–0.057	0.635	—	
t0 diff	0.126	–0.181	–0.024	–0.061	–0.375	0.310	–0.793	–0.183	—

The results showed a replication of the large negative correlation between *response caution*-difference and *t0*-difference as found in Simulation 1, suggesting that our main finding is reproducible across different theoretical implementations of evidence-accumulation models. In addition, we again found a (large) positive correlation between *caution*-difference and *v*-difference, and a (small) negative correlation between *v*-difference and *t0*-difference.

### Simulation 5: True Correlated Difference Scores

So far, the Simulations have examined cases where no true correlation exists between difference scores in the diffusion model parameters. We have shown under such circumstances, a spurious correlation emerges between the *a*-difference and *t0*-difference parameters. In the current simulation, we examine the complement of this whereby we simulate artificial data where a true correlation exists between *a*-difference and *t0*-difference parameters. The question we wished to address is whether the model fitting procedure is able to recover such a true correlation, or whether recovery is corrupted by whatever is causing the spurious correlations reported in previous simulations^5^.

#### Recovery of a true negative correlation

In the first simulation in this section, we explored recovery attempts from a simulated data set with a true population-level correlation of *r* = – .7 between the *a*-difference and *t0*-difference parameter values. We simulated data consisting of 1000 trials for 1000 artificial participants in two conditions (“easy” and “hard”). The method used to generate the parameters that would be used to simulate the data for each participant in the easy condition was the same as in Simulation 1. The method used to generate the parameters that would be used to simulate the data in the hard condition was as follows:

$${\displaystyle \begin{array}{c}{v}_{\mathrm{hard}}={v}_{\mathrm{easy}}\\ {}\begin{array}{c}{a}_{\mathrm{hard}}={a}_{\mathrm{easy}}+{a}_{\mathrm{dif}\mathrm{f}}\\ {}t{0}_{\mathrm{hard}}=t{0}_{\mathrm{easy}}+t{0}_{\mathrm{dif}}\end{array}\end{array}}$$

That is, the drift rate was identical for the easy and the hard condition. The boundary separation parameter in the hard condition was generated by taking the boundary separation parameter in the easy condition and adding a difference score to it (*a*_diff_). The non-decision time parameter in the hard condition was generated by taking the non-decision time parameter in the easy condition and adding a difference score to it (*t0*_diff_). The difference scores *a*_diff_ and *t0*_diff_ were sampled from a multivariate normal distribution with mean *a*_diff_ set to zero, SD *a*_diff_ set to 0.1, mean *t0*_diff_ set to zero, and SD *t0*_diff_ set to 0.05. The population correlation between *a*_diff_ and *t0*_diff_ was set to *r* = – 0.7. Put another way, the population-level difference in boundary separation and non-decision time between the hard and the easy condition was zero, but there was a true correlation in difference scores between *a*_diff_ and *t0*_diff_ of *r* = – 0.7. The parameter fitting routine was the same as in Simulation 1.

The results of the Simulation can be seen in Table 9. Critically, the “true” negative correlation between *a*_diff_ and *t0*_diff_ was relatively well recovered (*r* = – 0.673).

**Table 9:** Product-moment correlation coefficients between the fitted parameters from the “true negative” correlation in Simulation 5. Diff = difference scores on parameters (hard minus easy)

	*a* (easy)	*a* (hard)	*v* (easy)	*v* (hard)	*t0* (easy)	*t0* (hard)	*a* diff	*v* diff	*t0* diff
*a* (easy)	—								
*a* (hard)	0.996	—							
*v* (easy)	0.007	– 0.005	—						
*v* (hard)	0.003	– 0.001	0.994	—					
*t0* (easy)	0.006	0.020	– 0.012	– 0.011	—				
*t0* (hard)	0.009	– 0.072	0.031	0.028	0.848	—			
*a* diff	– 0.023	0.237	– 0.044	– 0.017	0.052	– 0.312	—		
*v* diff	– 0.039	0.029	0.002	0.109	0.010	– 0.027	0.256	—	
*t0* diff	0.007	– 0.168	0.077	0.069	– 0.036	0.500	– 0.673	– 0.066	—

#### Recovery of a true positive correlation

In the next simulation in this section, we repeated the previous simulation but simulated data so that the true population-level correlation between *a*_diff_ and *t0*_diff_ was set to 0.7. The results of this simulation can be seen in Table 10. The “true” positive correlation between *a*_diff_ and *t0*_diff_ was relatively well recovered (*r* = 0.521). Although there is some discrepancy between the recovered correlation and the “true” generating correlation, this likely reflects sampling error and/or simulation noise.

**Table 10:** Product-moment correlation coefficients between the fitted parameters from the “true positive” correlation in Simulation 5. Diff = difference scores on parameters (hard minus easy)

	*a* (easy)	*a* (hard)	*v* (easy)	*v* (hard)	*t0* (easy)	*t0* (hard)	*a* diff	*v* diff	*t0* diff
*a* (easy)	—								
*a* (hard)	0.969	—							
*v* (easy)	0.004	0.000	—						
*v* (hard)	–0.005	–0.003	0.994	—					
*t0* (easy)	0.009	0.027	–0.006	–0.002	—				
*t0* (hard)	0.004	0.084	0.015	0.015	0.868	—			
*a* diff	–0.036	0.211	–0.016	0.009	0.075	0.323	—		
*v* diff	–0.076	–0.021	–0.081	0.033	0.039	–0.003	0.216	—	
*t0* diff	–0.006	0.122	0.041	0.033	0.006	0.502	0.521	–0.075	—

In general, though, the results of Simulation 5 show that if a true correlation exists between a_difference_ and t0_difference_, the model fitting procedures of the diffusion model are able to recover this correlation relatively well. Parameter recovery in situations with true correlations between difference scores is therefore not corrupted by whatever is causing the spurious correlations reported in previous simulations.

## General discussion

The use of evidence-accumulation models such as the drift-diffusion model hold great promise in addressing the so-called “reliability paradox” (Hedge et al., 2018; Rouder & Haaf, 2019): By analysing individual differences at the latent level (e.g., via DDM parameters) researchers can uncover relationships not apparent at the purely behavioural level. The current work was inspired by an incidental observation that when difference scores of DDM parameters were analysed, a large and negative correlation emerged between the difference score in the boundary separation parameter and the difference score in the non-decision time parameter. In this paper we have confirmed this finding more formally, via reanalysis of behavioural data (the two experiments of Schuch & Grange, 2019, as well as three experiments from Dutilh et al., 2019) and via a series of computational simulation studies. These simulations were conducted across a range of software implementations of the DDM, as well as using a different evidence-accumulation model (the LBA; Brown & Heathcote, 2008). In Appendix C we outline details of additional analyses to explore whether the spurious correlation is caused by difficulties in parameter optimisation when accuracy rates are very high, but find that it had little impact. In addition, in Appendix D we report the results of fitting a Bayesian hierarchical version of the diffusion model (as implemented in the *hBayesDM* package in R; Ahn et al., 2017) to Experiment 1 data of Dutilh et al. (2019) and again find a large negative correlation. Together, these results using different models and fitting techniques suggest that the result is general.

We begin this discussion by summarising our behavioural and simulation findings; we then offer some suggestions as to the potential cause of the spurious correlation between boundary separation difference and non-decision time difference, before providing some recommendations for researchers wishing to use DDM or LBA difference scores in their own work.

### Summary of findings

#### Correlation between a-difference and t0-difference

The correlation between *a*-difference and *t0*-difference emerged in the majority of our analyses. Specifically, it was present in all of the experiments and conditions reanalysed from Schuch and Grange (2019), was present in two out of three experiments in Dutilh et al. (2019), and was present in the simulations (but not all; see later). Visualisation of this correlation in the Schuch and Grange (2019) data (Fig. 2) and in the simulations where it was present (Fig. 4) suggest this negative correlation is not driven by outliers in the data, but indeed represents a strong negative linear relationship across the range of difference scores. In the simulations, we only observed the negative correlation when either (a) there was no true difference in model parameters between simulated experimental conditions, or (b) only drift rate *v* was manipulated between simulated experimental conditions; when a true difference existed in boundary separation, non-decision time, or all three main parameters, the correlation disappeared. This suggests that the spurious correlation occurs only when there is no difference between experimental conditions, or the difference is isolated to the drift rate. This simulation result is congruent with the reanalysis of the behavioural data. For example, Experiment 1 of Dutilh et al. (2019) had no behavioural manipulation between the two experimental conditions, and we observed the negative correlation between *a*-difference and *t0*-difference; in Experiment 2, the behavioural manipulation influenced estimates of drift rate, and again we observed the negative correlation; in Experiment 3, however, the behavioural manipulation influenced the boundary separation parameter, and the negative correlation between *a*-difference and *t0*-difference disappeared.

#### Further correlations between parameter difference scores

While the negative correlation between *boundary*-difference and *non-decision*-difference was the most pronounced and most consistent relationship between parameter difference scores, we also observed some other correlations between parameter difference scores. In particular, we observed medium-to-large correlations between *a*-difference and *v*-difference in the reanalysis of Experiment 1 of Schuch and Grange (2019; ranging from *r* = 0.33 to 0.54) and in Experiment 1 of Dutilh et al. (2019; *r* = 0.30); this correlation was absent from the reanalysis of Experiment 2 of Schuch and Grange, but was medium and *negative* in Experiments 2 and 3 of Dutilh et al. (*r*s *=* – 0.32 and – 0.22, respectively). This correlation emerged also in Simulation 1 using the DDM (*r* = 0.47), and in Simulation 4 using the LBA (*r* = 0.64). Recall that these simulations had no true difference in parameter values between conditions. In Simulation 2 where changes in parameters were introduced, we observed the correlation between *a*-difference and *v*-difference only when *t0* was selectively changed (ranging from *r* = 0.46 to .052). When other parameters were selectively changed between conditions, or all three parameters were changed, the correlation disappeared.

This correlation arises due to individuals with larger difference scores in boundary separation having larger difference scores in drift rate; but note that if the difference score in drift rate is positive, this reflects *higher* drift rates in the *hard* condition relative to the *easy* condition. This is because the difference score is calculated as the parameter estimate for the *hard* condition minus the parameter estimate for the *easy* condition, and drift rates tend to be *lower* in harder conditions than in easy conditions. One possibility is that there is some form of trade-off occurring during the model fitting when no true differences exist between conditions in the boundary separation and drift rate parameters.

We sometimes observed a correlation between *v*-difference and *t0*-difference. Although not consistently present, it was small-to-medium and positive in Experiment 1 Conditions 2 and 4 (*r*s = 0.18 & 0.30 respectively) and Experiment 2 Conditions 1 and 2 of Schuch and Grange (*r*s = 0.16 and 0.41, respectively), as well as in Experiments 2 and 3 in Dutilh et al. (2019; *r*s = 0.44 and 0.38, respectively); it was small and negative in Experiment 1 of Dutilh et al. (*r* = – 0.17). In Simulations 1 and 4, this correlation was small and negative (*r*s = – 0.22 & – 0.24, respectively), but note that it did not replicate in the EZ-diffusion analysis of the same data in Simulation 1. It was however again present in the LBA model in Simulation 4 (*r* = – 0.18). It was also present in Simulation 2, but only when boundary separation was manipulated between conditions (r*s* = – 0.39, – 0.17, & – 0.22 for small, medium, and large effect size differences, respectively). Hence, from the simulation studies, it seems that a small negative spurious correlation between *v*-difference and *t0*-difference emerges when the third parameter, boundary separation, is manipulated between conditions, or when there are no differences between the conditions at all.

In general, the simulation results seem to suggest that when there is a difference between conditions in one of the main parameters (or no difference between conditions at all), a spurious correlation tends to occur between the difference scores of the other two parameters. The size of the spurious correlation between difference scores is largest for the relationship between *a*-difference and *t0*-difference, and medium-to-small for the relationship between *v*-difference and *a*-difference, and *v*-difference and *t0*-difference.

### Recommendations for researchers

In all of the simulations reported in the paper (and indeed the model fits to behavioural data), we allowed all three main parameters to freely vary across experimental conditions. Such an approach is common in the literature when wishing to draw inferences on meaningful parameter changes across conditions (see Donkin et al., 2011; Dutilh et al., 2019). However, it could be that the spurious correlation between *a*-difference and *t0*-difference is exacerbated by unnecessary model flexibility (i.e., when parameters are allowed to vary freely across conditions even when there was no true parameter difference in the data-generating process). To assess this, we conducted an additional simulation similar to the design of Simulation 1 where no true difference exists between two experimental conditions in the generated data; however, during model fitting, drift rate—although still a free parameter—was constrained to take on the same value across conditions. The correlations between best-fitting parameters using *fast-dm-30* are shown in Table 11. The correlation between *a*-difference and *t0*-difference was still large and negative. Of course, this model is still more flexible than the “true” data-generating model, but constraining boundary separation and/or non-decision time to be equal across conditions would prohibit us from examining the difference score in these parameters.

**Table 11:** Product-moment correlation coefficients between the fitted parameters from the fast-dm-30 fitting routine with drift rate not free to vary between conditions. Diff = difference scores on parameters (hard minus easy)

	*a* (easy)	*a* (hard)	*v*	*t0* (easy)	*t0* (hard)	*a* diff	*t0* diff
*a* (easy)	—						
*a* (hard)	0.994	—					
*v*	0.042	0.036	—				
*t0* (easy)	– 0.047	– 0.043	0.004	—			
*t0* (hard)	– 0.041	– 0.042	0.006	0.998	—		
*a* diff	– 0.090	0.016	– 0.058	0.037	– 0.011	—	
*t0* diff	0.096	0.016	0.029	– 0.068	– 0.003	– 0.752	—

This leads us to a recommendation for researchers wishing to use evidence-accumulation models in assessments of individual differences: Only allow parameters to vary freely across conditions during the fitting process if there is strong a priori theoretical justification for supposing parameter differences are expected, or formal model selection/competition techniques have suggested that models with additional free parameters are justified (Donkin et al., 2011; see Heathcote et al., 2015 for an excellent overview of this and other general recommendations to researchers using formal modelling). Formal model selection techniques, such as Bayesian information criterion (BIC) and Akaike information criterion (AIC), can be used to temper quality of model fit with a penalty term for additional free parameters: All else being equal, if two models fit equally well, the simpler model—that is, the model with fewer free parameters—will be preferred. With this approach, difference scores on certain model parameters will only be calculated by the researcher when the model-selection process has suggested that a model where these certain model parameters change across conditions fits the data better than a model where the parameters are fixed across conditions. This would then avoid the spurious correlation reported in the current paper as we do not find the spurious correlation between *a*-difference and *t0*-difference when the true data-generating model had real differences in either of these parameters across conditions.

To exemplify the technique of model competition, and how it would avoid the calculation of a spurious correlation between *a*-difference and *t0-*difference, we conducted formal model selection techniques on the data generated in Simulation 1, where no true differences in parameter values existed across conditions in the data-generating process. Eight possible models were then constructed (see Table 12); each model differed on the eligibility of one or more DDM parameters to freely vary across conditions. The simplest model—Model 1, which has only three free parameters (*v*, *a*, and *t0*, which are not free to vary across conditions)—is correctly selected by the model selection technique of choosing the model with the lowest AIC value (or BIC value).

**Table 12:** Results of the model competition approach to analysing data generated from Simulation 1. Each row depicts a different model based on whether drift rate (*v*), boundary separation (*a*), and non-decision time (*t0*) is free to vary across conditions or not (if yes, denoted by a tick, and by a cross if not). LL is the log-likelihood of the fit. AIC and BIC refer to the total Akaike and Bayesian information criteria, respectively, summed across all simulated participants. W_AIC_ and W_BIC_ represent Akaike weights for each model based on the AIC and BIC values, respectively. Bold & underlined model represents the winner of the model competition

Model	Vary *v*?	Vary *a*?	Vary *t0*?	LL	AIC	BIC	W_AIC_	W_BIC_
Model 1	**x**	**x**	**x**	**– 715,490**	**1,436,982**	**1,453,784**	**1**	**1**
Model 2	✓	x	x	– 715,956	1,439,912	1,462,316	0	0
Model 3	x	✓	x	– 715,946	1,439,893	1,462,296	0	0
Model 4	x	x	✓	– 716,093	1,440,185	1,462,589	0	0
Model 5	x	✓	✓	– 716,684	1,443,367	1,471,372	0	0
Model 6	✓	x	✓	– 716,637	1,443,274	1,471,279	0	0
Model 7	✓	✓	x	– 716,401	1,442,803	1,470,807	0	0
Model 8	✓	✓	✓	– 717,127	1,446,253	1,479,858	0	0

In this case, the researcher would not be justified in interpreting a model where *a* and *t0* are free to vary across conditions, thus avoiding the spurious correlation between *a*-difference and *t0*-difference. In addition, note that in Simulation 2 where the data were generated from a “true model” with all three main parameters changing across conditions we did not observe the spurious negative correlation (see Fig. 3). In this situation, a model where all three parameters are free to vary would win the model competition, and the spurious correlation would likely not be present in any subsequent individual differences analysis.

However, recall that in Simulation 5 we generated simulated data with no true difference between any of the main parameters between conditions but with a large correlation between *a*-difference and *t0*-difference. We were interested in whether this correlation could be recovered by the model-fitting procedure, and found that it could. However, as no true difference was present between any of the three main parameters across conditions, model competition techniques select a model in which the main parameters are not allowed to freely vary across conditions (see Appendix E); as such, researchers would miss the true correlation between *a*-difference and *t0*-difference because they would fit a model to the data where these parameters do not change across condition (so there are no difference scores in parameters).

The recommendation we provide to engage in model competition appears to minimise exposure to the spurious correlation—which likely arises only in overly flexible models—but also leaves the possibility that a true correlation between a-difference and t0-difference could be missed if the best-fitting model is one where the parameters are not free to vary across conditions. Note, however, that such a situation where there is a true correlation between the *a*-difference and *t0*-difference—but at the same time the mean difference across participants in both parameters is zero—probably does not occur very often in real data. In summary, we believe the cost of finding a spurious correlation outweighs the cost of missing a true correlation and recommend engaging in model competition techniques and only analysing further the winning model.

An additional tool to use for model selection is Akaike weights (e.g., Wagenmakers & Farrell, 2004). Selecting a model based on raw AIC or BIC scores becomes challenging when two (or more) models’ IC scores differ by a small amount. Although one can still select the model with the smallest IC score, the researcher is left wondering how strongly they can disregard the next-best model. Akaike weights estimates the probability that each model in the set of all models considered will be superior when applied to new data (McElreath, 2020). As such, these weights can be used to quantify the degree of superiority of the winning model. As can be seen in Table 12, the winning model has a weight of 1, indicating decisive support for this model. For more information and further use-cases of Akaike weights, see Wagenmakers and Farrell (2004). Importantly, both of the model selection techniques discussed—that based on overall AIC or BIC values, and that based on Akaike weights—can be applied to group-averaged data (as above) or applied to the individual participant level. A model that is superior at the group level may not be superior for each participant.

As an alternative (or complement) to formal model selection, researchers could use a structural equation modelling approach for estimating the parameters of evidence-accumulation models (e.g., Schubert et al., 2022)^6^. In such a framework, the evidence-accumulation model parameters can be conceptualised as latent variables (which are estimated repeatedly on the basis of different subsets of the raw data; for example, separate estimations on the basis of odd-numbered and even-numbered trials). The difference scores in evidence-accumulation model parameters can then be estimated as latent change scores, which are derived by regressing the parameter estimates from the “hard” condition on the parameter estimates from the “easy” condition (for reviews of latent change score modelling see Kievit et al., 2018 and McArdle, 2009). The latent change scores representing the difference scores can then be correlated. In Appendix F, we report first investigations of the spurious correlations with latent change score models. We observed a Heywood case: small and non-significant variances of latent change scores, but large correlations between change scores, including a large negative correlation between the latent change score for boundary separation and non-decision time (i.e., the spurious correlation). However, we found that in models where correlations could occur between indicator diffusion model parameters within a condition, the spurious correlation was absent. This provides preliminary evidence that the correlations of model parameters within a condition could play a role for the spurious correlations between difference scores to occur and that latent change score modelling might be a useful approach to mitigate its impact.

## Conclusions

We have identified—both in behavioural data and via computational simulations—that spurious correlations can arise between model parameter difference scores when using evidence-accumulation models. Researchers wishing to use this class of models for inter-individual differences research should be aware of these spurious correlations, and should bear them in mind when wishing to draw conclusions from difference-scores. One way we have identified to mitigate the impact of these spurious correlations—but we submit that there will be other solutions we have not thought of—is to conduct formal model competition to ensure that difference scores are only calculated for a model parameter when model competition has shown that such parameter flexibility is warranted by the data.

### Supplementary information

ESM 1 (DOCX 3.23 MB)

## Footnotes

1 Precision values control the accuracy of the calculated density functions in *fast-dm-30*. Higher values lead to higher accuracy of prediction but leads to slower simulation time. Voss et al. (2015) recommend setting *precision* to values between 2.0 and 5.0.

2 Usually, trial-to-trial variability in non-decision time (implemented in *fast-dm-30* via the *st0* parameter) is not fixed to zero to account for very rapid responses often present in real data sets (Voss et al., 2015). We repeated Simulation 1 with *st0* allowed to be a free parameter during the model fitting and found qualitatively identical results to those reported in the text body. All simulations therefore fixed *st0* to zero, which also has the benefit of reducing simulation time.

3 As the EZ diffusion model is a closed-form solution where the main DDM parameters are calculated from mean RT, RT variance, and percentage accuracy, edge corrections are required when accuracy is at 100, 50, or 0%. We used the same correction as reported by Lerche et al. (2017): When accuracy was 100%, we set accuracy equal to 100 – 100 / (2n), where n is the number of trials used in the simulation (here *n* = 1000); when accuracy was 50%, it was corrected to 50 + 100 / (2n), and to 100 / (2n) when accuracy was 0%.

4 Note that although the rtdists package implements the drift-diffusion model in R, it is based on C code underlying the fast-dm-30 software. As the purpose of Simulation 3 was to make sure our results in Simulation 1 and Simulation 2 were not due to peculiarities in the simulation and/or fitting algorithms in fast-dm-30, it might therefore seem problematic to use *rtdists*. However, the code for *rtdists* has been adapted to C++ by Singmann et al. (2020), which then is called by functions written in R. Thus we consider this to be an independent implementation of the drift-diffusion model and is therefore suitable for our purposes.

5 We are grateful to an anonymous reviewer for suggesting this simulation study.

6 We would like to thank Anna-Lena Schubert for this idea.

## References

[CR1] Ahn W-Y, Haines N, Zhang L (2017). Revealing neurocomputational mechanisms of reinforcement learning and decision-making with the hBayesDM package. Computational Psychiatry.

[CR2] Boehm U, Annis J, Frank MJ, Hawkins GE, Heathcote A, Kellen D, Krypotos A-M, Lerche V, Logan GD, Palmeri TJ, van Ravenzwaaij D, Servant M, Singmann H, Starns JJ, Voss A, Wiecki TV, Matzke D, Wagenmakers E-J (2018). Estimating across-trial variability parameters of the Diffusion Decision Model: Expert advice and recommendations. Journal of Mathematical Psychology.

[CR3] Borsboom D, Kievit R, Cervone D, Hood S, Valsiner J, Molenaar P, Lyra M, Chaudhary N (2009). The Two Disciplines of Scientific Psychology, or: The Disunity of Psychology as a Working Hypothesis. *Dynamic Process Methodology in the Social and Developmental Sciences*.

[CR4] Brown SD, Heathcote A (2008). The simplest complete model of choice response time: Linear ballistic accumulation. Cognitive Psychology.

[CR5] Cronbach LJ (1957). The two disciplines of scientific psychology. American Psychologist.

[CR6] Donkin C, Brown S, Heathcote A (2011). Drawing conclusions from choice response time models: A tutorial using the linear ballistic accumulator. Journal of Mathematical Psychology.

[CR7] Durst M, Janczyk M (2019). Two types of Backward Crosstalk: Sequential modulations and evidence from the diffusion model. Acta Psychologica.

[CR8] Dutilh, G., Annis, J., Brown, S. D., Cassey, P., Evans, N. J., Grasman, R. P. P. P., Hawkins, G. E., Heathcote, A., Holmes, W. R., Krypotos, A.-M., Kupitz, C. N., Leite, F. P., Lerche, V., Lin, Y.-S., Logan, G. D., Palmeri, T. J., Starns, J. J., Trueblood, J. S., van Maanen, L., … Donkin, C. (2019). The quality of response time data inference: A blinded, collaborative assessment of the validity of cognitive models. *Psychonomic Bulletin & Review*, *26*, 1051–1069. 10.3758/s13423-017-1417-2 10.3758/s13423-017-1417-2 PMC6449220 29450793

[CR9] Euler MJ, Schubert A-L (2021). Recent developments, current challenges, and future directions in electrophysiological approaches to studying intelligence. Intelligence.

[CR10] Forstmann BU, Wagenmakers EJ, Eichele T, Brown S, Serences JT (2011). Reciprocal relations between cognitive neuroscience and formal cognitive models: Opposites attract?. Trends in Cognitive Sciences.

[CR11] Forstmann BU, Ratcliff R, Wagenmakers EJ (2016). Sequential sampling models in cognitive neuroscience: Advantages, applications, and extensions. Annual Review of Psychology.

[CR12] Gade M, Schuch S, Druey M, Koch I, Grange J, Houghton G (2014). Inhibitory control in task switching. Task switching and cognitive control.

[CR13] Heathcote A, Brown SD, Wagenmakers E-J, Forstmann BU, Wagenmakers E-J (2015). An introduction to good practices in cognitive modeling. *An Introduction to Model-Based Cognitive Neuroscience*.

[CR14] Heathcote A, Lin Y-S, Reynolds A, Strickland L, Gretton M, Matzke D (2019). Dynamic models of choice. Behavior Research Methods.

[CR15] Hedge C, Powell G, Sumner P (2017). The reliability paradox: Why robust cognitive tasks do not produce reliable individual differences. Behavior Research Methods.

[CR16] Hedge C, Powell G, Bompas A, Vivian-Griffiths S, Sumner P (2018). Low and variable correlation between reaction time costs and accuracy costs explained by accumulation models: Meta-analysis and simulations. Psychological Bulletin.

[CR17] Hedge C, Vivian-Griffiths S, Powell G, Bompas A, Sumner P (2019). Slow and steady? Strategic adjustments in response caution are moderately reliable and correlate across tasks. Consciousness and Cognition.

[CR18] Hedge, C., Powell, G., Bompas, A., & Sumner, P. (2021). Strategy and processing speed eclipse individual differences in control ability in conflict tasks. *Journal of Experimental Psychology: Learning, Memory & Cognition*. Manuscript accepted for publication. Preprint available at https://www.psyarxiv.com/vgpxq/. 10.1037/xlm0001028 PMC9899369 34591554

[CR19] Heitz RP (2014). The speed–accuracy tradeoff: History, physiology, methodology, and behavior. Frontiers in Neuroscience.

[CR20] Janczyk M, Lerche V (2019). A Diffusion Model Analysis of the Response-Effect Compatibility Effect. Journal of Experimental Psychology: General.

[CR21] Janczyk M, Büschelberger J, Herbort O (2017). Larger between-task crosstalk in children than in adults: Behavioral results from the backward crosstalk paradigm and a diffusion model analysis. Journal of Experimental Child Psychology.

[CR22] Kievit RA, Brandmaier AM, Ziegler G, van Harmelen A-L, de Mooij SMM, Moutoussis M, Goodyer IM, Bullmore E, Jones PB, Fonagy P, Lindenberger U, Dolan RJ (2018). Developmental cognitive neuroscience using latent change score models: A tutorial and applications. Developmental Cognitive Neuroscience.

[CR23] Koch I, Gade M, Schuch S, Philipp AM (2010). The role of inhibition in task switching: A review. Psychonomic Bulletin & Review.

[CR24] Kowalczyk AW, Grange JA (2020). The effect of episodic retrieval on inhibition in task switching: A diffusion model analysis. Psychological Research.

[CR25] Lerche L, Voss A (2017). Retest reliability of the parameters of the Ratcliff diffusion model. Psychological Research.

[CR26] Lerche V, Voss A, Nagler M (2017). How many trials are required for parameter estimation in diffusion modeling? A comparison of different optimization criteria. Behavior Research Methods.

[CR27] Lerche V, von Krause M, Voss A, Frischkorn GT, Schubert A-L, Hagemann D (2020). Diffusion modeling and intelligence: Drift rates show both domain-general and domain-specific relations with intelligence. Journal of Experimental Psychology: General.

[CR28] Mayr U, Gorfein DS, MacLeod CM (2007). Inhibition of task sets. Inhibition in cognition.

[CR29] Mayr U, Keele SW (2000). Changing internal constraints on action: The role of backward inhibition. Journal of Experimental Psychology: General.

[CR30] McArdle JJ (2009). Latent variable modeling of differences and changes with longitudinal data. Annual Review of Psychology.

[CR31] McElreath R (2020). *Statistical rethinking: A Bayesian course with examples in R and Stan*.

[CR32] Miller J, Ulrich R (2013). Mental chronometry and individual differences: Modeling reliabilities and correlations of reaction time means and effect sizes. Psychonomic Bulletin & Review.

[CR33] Parsons S, Kruijt A-W, Fox E (2019). Psychological science needs a standard practice of reporting the reliability of cognitive-behavioral measurements. Advances in Methods and Practices in Psychological Science.

[CR34] Rae B, Heathcote A, Donkin C, Averell L, Brown S (2014). The hare and the tortoise: Emphasizing speed can change the evidence used to make decisions. *Journal of Experimental Psychology*. Learning, Memory, and Cognition.

[CR35] Ratcliff R, Childers R (2015). Individual differences and fitting methods for the two-choice diffusion model. Decision.

[CR36] Ratcliff R, McKoon G (2008). The diffusion decision model: Theory and data for two-choice decision tasks. Neural Computation.

[CR37] Ratcliff R, Smith PL, Brown SD, McKoon G (2016). Diffusion decision model: Current issues and history. Trends in Cognitive Sciences.

[CR38] Rouder JN, Haaf JM (2019). A psychometrics of individual differences in experimental tasks. Psychonomic Bulletin & Review.

[CR39] Rouder, J. N., Kumar, A., & Haaf, J. M. (2019). Why most studies of individual differences with inhibition tasks are bound to fail. Preprint retrieved from: 10.31234/osf.io/3cjr5

[CR40] Schmitz F, Voss A (2012). Decomposing task-switching costs with the diffusion model. Journal of Experimental Psychology: Human Perception and Performance.

[CR41] Schmitz F, Voss A (2014). Components of task switching: A closer look at task switching and cue switching. Acta Psychologica.

[CR42] Schubert A-L, Hagemann D, Voss A, Schankin A, Bergmann K (2015). Decomposing the relationship between mental speed and mental abilities. Intelligence.

[CR43] Schubert A-L, Frischkorn GT, Hagemann D, Voss A (2016). Trait Characteristics of Diffusion Model Parameters. Journal of Intelligence.

[CR44] Schubert A-L, Ferreira MB, Mata A, Riemenschneider B (2021). A diffusion model analysis of belief bias: Different cognitive mechanisms explain how cognitive abilities and thinking styles contribute to conflict resolution in reasoning. Cognition.

[CR45] Schubert, A.-L., Löffler, C., & Hagemann, D. (2022). A neurocognitive psychometrics account of individual differences in attentional control. *Journal of Experimental Psychology: General*. Advance online publication. 10.1037/xge0001184 10.1037/xge0001184 35130011

[CR46] Schuch S (2016). Task inhibition and response inhibition in older versus younger adults: A diffusion model analysis. Frontiers in Psychology.

[CR47] Schuch S, Grange JA (2019). Increased cognitive control after task conflict? Investigating the N-3 effect in task switching. Psychological Research.

[CR48] Schuch S, Konrad K (2017). Investigating Task Inhibition in Children versus Adults: A diffusion model analysis. Journal of Experimental Child Psychology.

[CR49] Servant M, Montagnini A, Burle B (2014). Conflict tasks and the diffusion framework: Insight in model constraints based on psychological laws. Cognitive Psychology.

[CR50] Singmann, H., Brown, S., Gretton, M., & Heathcote, A. (2020). rtdists: Response time distributions. R package version 0.11-2. Retrieved from https://cran.r-project.org/web/packages/rtdists/index.html

[CR51] Voss A, Voss J (2007). Fast-dm: a free program for efficient diffusion model analysis. Behavior Research Methods.

[CR52] Voss A, Voss J, Lerche V (2015). Assessing cognitive processes with diffusion model analyses: A tutorial based on fast-dm-30. Frontiers in Psychology.

[CR53] Wagenmakers E-J, Farrell S (2004). AIC model selection using Akaike weights. Psychonomic Bulletin & Review.

[CR54] Wagenmakers E-J, van der Maas HLJ, Grasman RPPP (2007). An EZ-diffusion model for response time and accuracy. Psychonomic Bulletin & Review.

[CR55] White CN, Servant M, Logan GD (2018). Testing the validity of conflict drift-diffusion models for use in estimating cognitive processes: A parameter-recovery study. Psychonomic Bulletin & Review.

