# Supplementary material for: A spurious correlation between difference scores in evidence-accumulation model parameters
Source: Behav Res Methods. 2022 Sep 22;55(7):3348–69. doi: 10.3758/s13428-022-01956-8 (PMC10615941; doi:10.3758/s13428-022-01956-8)
Supplement: Supplementary file 1 — (DOCX 3.23 MB) [file 13428_2022_1956_MOESM1_ESM.docx]

**Appendices**

**Appendix A—Parameter Recovery Visualisations for Simulation 1**

**Appendix B—Simulation 2 Numerical Correlation Matrices**

**Appendix C—Examining the Impact of High Accuracy**

**Appendix D—Bayesian Hierarchical Diffusion Modelling**

**Appendix E—Model Competition for Simulation 5**

**Appendix F—Latent Change Score Modelling**

**Appendix A—Parameter Recovery Visualisations for Simulation 1**

Recovery of the generating parameter values used to generate simulated data was excellent in the fitting routine. The correlation between the parameter values used to generate simulated data and the recovered best-fitting parameter values were as follows: *a*-easy: *r* = .995, *t0*-easy: *r* = .999, *v*-easy: *r* = .997, *a*-hard: *r* = .996, *t0*-hard: *r* = .999, *v*-hard: *r* = .997.

The correlations are visualised in Figure A1.

**
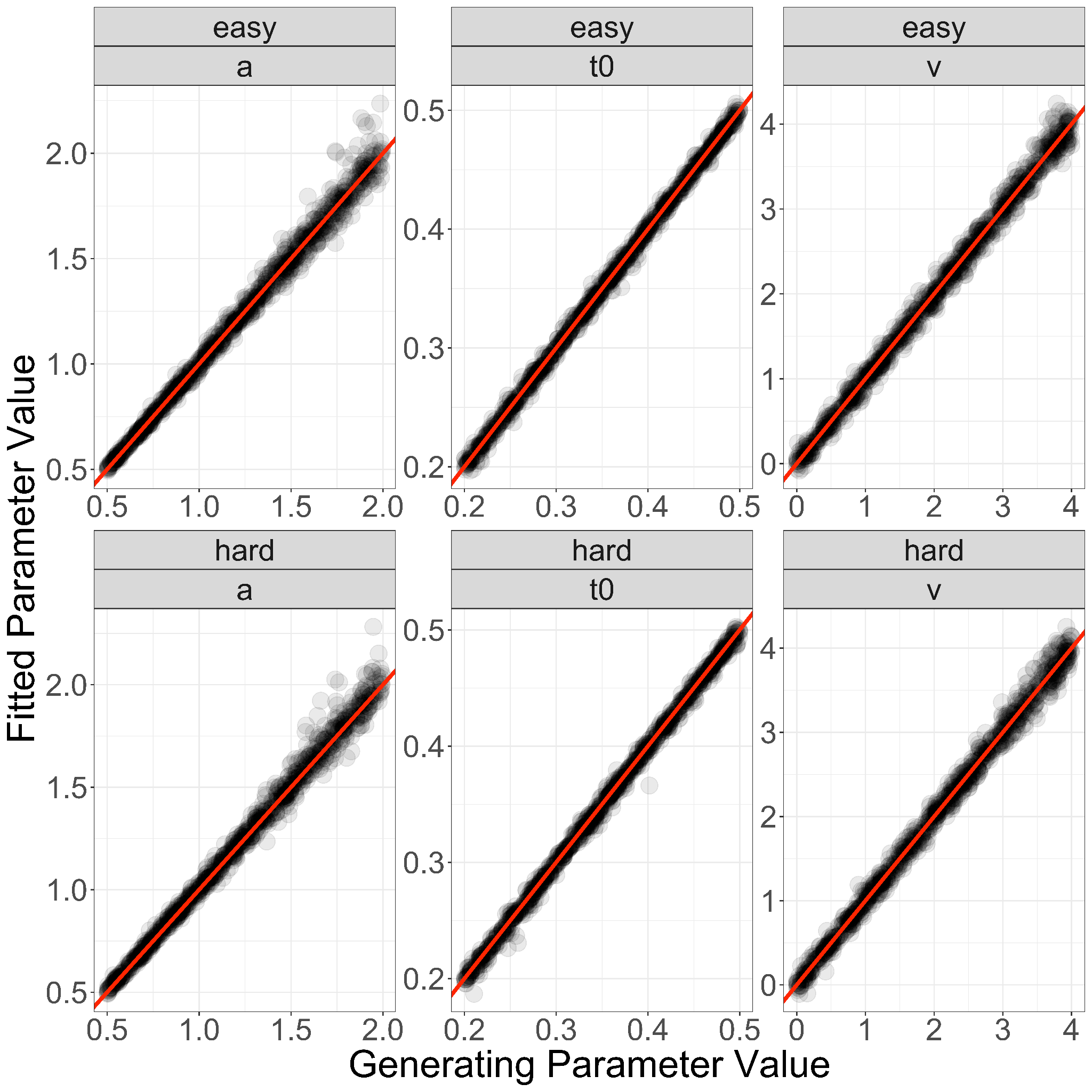
**

**Figure A1.** Visualisation of correspondence between parameter values used to generate simulated data (x-axis) and the recovered best-fitting parameter value (y-axis) for each simulated participant (shown as grey circles). Results are shown for the three main diffusion model parameters in both the “easy” and the “hard” condition. The diagonal red lines correspond to location representing perfect recovery.

**Appendix B—Simulation 2 Correlation Matrices**

**v Small**

| **Table B1.** Product-moment correlation coefficients between the fitted parameters from fast-dm-30 fitting routine from Simulation 2 where drift rate (v) was manipulated between easy and hard conditions to have a small effect size. | | | | | | | | | |
| --- | --- | --- | --- | --- | --- | --- | --- | --- | --- |
|  | **a (easy)** | **a (hard)** | **v**  **(easy)** | **v (hard)** | **t0 (easy)** | **t0 (hard)** | **a**  **diff** | **v**  **diff** | **t0**  **diff** |
| **a (easy)** | — |  |  |  |  |  |  |  |  |
| **a (hard)** | 0.991 | — |  |  |  |  |  |  |  |
| **v (easy)** | 0.004 | -0.006 | — |  |  |  |  |  |  |
| **v (hard)** | -0.033 | -0.033 | 0.517 | — |  |  |  |  |  |
| **t0 (easy)** | -0.016 | 0.000 | -0.046 | -0.051 | — |  |  |  |  |
| **t0 (hard)** | -0.015 | -0.007 | -0.043 | -0.049 | 0.997 | — |  |  |  |
| **a diff** | -0.081 | 0.051 | -0.073 | -0.001 | 0.114 | 0.058 | — |  |  |
| **v diff** | -0.038 | -0.028 | -0.473 | 0.510 | -0.006 | -0.007 | 0.072 | — |  |
| **t0 diff** | 0.010 | -0.092 | 0.052 | 0.034 | -0.083 | -0.011 | -0.774 | -0.017 | — |

| **Table B2.** Product-moment correlation coefficients between the fitted parameters from EZ-diffusion fitting routine from Simulation 2 where drift rate (v) was manipulated between easy and hard conditions to have a small effect size. | | | | | | | | | |
| --- | --- | --- | --- | --- | --- | --- | --- | --- | --- |
|  | **a (easy)** | **a (hard)** | **v**  **(easy)** | **v (hard)** | **t0 (easy)** | **t0 (hard)** | **a**  **diff** | **v**  **diff** | **t0**  **diff** |
| **a (easy)** | — |  |  |  |  |  |  |  |  |
| **a (hard)** | 0.981 | — |  |  |  |  |  |  |  |
| **v (easy)** | -0.011 | -0.005 | — |  |  |  |  |  |  |
| **v (hard)** | -0.046 | -0.035 | 0.515 | — |  |  |  |  |  |
| **t0 (easy)** | -0.028 | 0.004 | -0.043 | -0.046 | — |  |  |  |  |
| **t0 (hard)** | -0.021 | -0.017 | -0.048 | -0.051 | 0.981 | — |  |  |  |
| **a diff** | -0.073 | 0.123 | 0.028 | 0.054 | 0.166 | 0.021 | — |  |  |
| **v diff** | -0.036 | -0.030 | -0.470 | 0.515 | -0.005 | -0.004 | 0.028 | — |  |
| **t0 diff** | 0.037 | -0.108 | -0.024 | -0.020 | -0.191 | 0.005 | -0.746 | 0.004 | — |

**a Small**

| **Table B3.** Product-moment correlation coefficients between the fitted parameters from fast-dm-30 fitting routine from Simulation 2 where boundary separation (a) was manipulated between easy and hard conditions to have a small effect size. | | | | | | | | | |
| --- | --- | --- | --- | --- | --- | --- | --- | --- | --- |
|  | **a (easy)** | **a (hard)** | **v**  **(easy)** | **v (hard)** | **t0 (easy)** | **t0 (hard)** | **a**  **diff** | **v**  **diff** | **t0**  **diff** |
| **a (easy)** | — |  |  |  |  |  |  |  |  |
| **a (hard)** | 0.487 | — |  |  |  |  |  |  |  |
| **v (easy)** | 0.058 | 0.051 | — |  |  |  |  |  |  |
| **v (hard)** | 0.047 | 0.058 | 0.993 | — |  |  |  |  |  |
| **t0 (easy)** | 0.011 | 0.074 | 0.030 | 0.029 | — |  |  |  |  |
| **t0 (hard)** | 0.014 | 0.067 | 0.033 | 0.028 | 0.996 | — |  |  |  |
| **a diff** | -0.491 | 0.521 | -0.006 | 0.011 | 0.063 | 0.054 | — |  |  |
| **v diff** | -0.092 | 0.060 | -0.026 | 0.089 | -0.007 | -0.043 | 0.150 | — |  |
| **t0 diff** | 0.028 | -0.077 | 0.035 | -0.010 | -0.013 | 0.080 | -0.104 | -0.391 | — |

| **Table B4.** Product-moment correlation coefficients between the fitted parameters from EZ-diffusion fitting routine from Simulation 2 where boundary separation (a) was manipulated between easy and hard conditions to have a small effect size. | | | | | | | | | |
| --- | --- | --- | --- | --- | --- | --- | --- | --- | --- |
|  | **a (easy)** | **a (hard)** | **v**  **(easy)** | **v (hard)** | **t0 (easy)** | **t0 (hard)** | **a**  **diff** | **v**  **diff** | **t0**  **diff** |
| **a (easy)** | — |  |  |  |  |  |  |  |  |
| **a (hard)** | 0.492 | — |  |  |  |  |  |  |  |
| **v (easy)** | 0.054 | 0.040 | — |  |  |  |  |  |  |
| **v (hard)** | 0.042 | 0.041 | 0.993 | — |  |  |  |  |  |
| **t0 (easy)** | -0.018 | 0.053 | 0.043 | 0.042 | — |  |  |  |  |
| **t0 (hard)** | 0.013 | 0.058 | 0.033 | 0.030 | 0.971 | — |  |  |  |
| **a diff** | -0.506 | 0.502 | -0.014 | -0.001 | 0.070 | 0.045 | — |  |  |
| **v diff** | -0.097 | 0.007 | -0.051 | 0.070 | -0.014 | -0.025 | 0.104 | — |  |
| **t0 diff** | 0.128 | 0.026 | -0.039 | -0.045 | -0.076 | 0.165 | -0.101 | -0.049 | — |

**t0 Small**

| **Table B5.** Product-moment correlation coefficients between the fitted parameters from fast-dm-30 fitting routine from Simulation 2 where non-decision time (t0) was manipulated between easy and hard conditions to have a small effect size. | | | | | | | | | |
| --- | --- | --- | --- | --- | --- | --- | --- | --- | --- |
|  | **a (easy)** | **a (hard)** | **v**  **(easy)** | **v (hard)** | **t0 (easy)** | **t0 (hard)** | **a**  **diff** | **v**  **diff** | **t0**  **diff** |
| **a (easy)** | — |  |  |  |  |  |  |  |  |
| **a (hard)** | 0.991 | — |  |  |  |  |  |  |  |
| **v (easy)** | 0.035 | 0.035 | — |  |  |  |  |  |  |
| **v (hard)** | 0.027 | 0.034 | 0.9994 | — |  |  |  |  |  |
| **t0 (easy)** | -0.037 | -0.031 | 0.018 | 0.014 | — |  |  |  |  |
| **t0 (hard)** | -0.006 | -0.011 | 0.011 | 0.003 | 0.497 | — |  |  |  |
| **a diff** | -0.063 | 0.070 | 0.000 | 0.056 | 0.045 | -0.035 | — |  |  |
| **v diff** | -0.069 | 0.000 | 0.013 | 0.123 | -0.035 | -0.068 | 0.514 | — |  |
| **t0 diff** | 0.031 | 0.020 | -0.007 | -0.011 | -0.493 | 0.510 | -0.079 | -0.034 | — |

| **Table B6.** Product-moment correlation coefficients between the fitted parameters from EZ-diffusion fitting routine from Simulation 2 where non-decision time (t0) was manipulated between easy and hard conditions to have a small effect size. | | | | | | | | | |
| --- | --- | --- | --- | --- | --- | --- | --- | --- | --- |
|  | **a (easy)** | **a (hard)** | **v**  **(easy)** | **v (hard)** | **t0 (easy)** | **t0 (hard)** | **a**  **diff** | **v**  **diff** | **t0**  **diff** |
| **a (easy)** | — |  |  |  |  |  |  |  |  |
| **a (hard)** | 0.979 | — |  |  |  |  |  |  |  |
| **v (easy)** | 0.043 | 0.039 | — |  |  |  |  |  |  |
| **v (hard)** | 0.036 | 0.039 | 0.994 | — |  |  |  |  |  |
| **t0 (easy)** | -0.048 | -0.038 | 0.014 | 0.006 | — |  |  |  |  |
| **t0 (hard)** | -0.013 | -0.029 | 0.003 | -0.003 | 0.496 | — |  |  |  |
| **a diff** | -0.087 | 0.119 | -0.022 | 0.016 | 0.050 | -0.082 | — |  |  |
| **v diff** | -0.062 | 0.006 | 0.004 | 0.117 | -0.064 | -0.054 | 0.331 | — |  |
| **t0 diff** | 0.035 | 0.008 | -0.011 | -0.009 | -0.4899 | 0.515 | -0.132 | 0.009 | — |

**v Medium**

| **Table B7.** Product-moment correlation coefficients between the fitted parameters from fast-dm-30 fitting routine from Simulation 2 where drift rate (v) was manipulated between easy and hard conditions to have a medium effect size. | | | | | | | | | |
| --- | --- | --- | --- | --- | --- | --- | --- | --- | --- |
|  | **a (easy)** | **a (hard)** | **v**  **(easy)** | **v (hard)** | **t0 (easy)** | **t0 (hard)** | **a**  **diff** | **v**  **diff** | **t0**  **diff** |
| **a (easy)** | — |  |  |  |  |  |  |  |  |
| **a (hard)** | 0.989 | — |  |  |  |  |  |  |  |
| **v (easy)** | -0.024 | -0.032 | — |  |  |  |  |  |  |
| **v (hard)** | -0.026 | -0.015 | 0.492 | — |  |  |  |  |  |
| **t0 (easy)** | 0.005 | 0.010 | -0.027 | 0.012 | — |  |  |  |  |
| **t0 (hard)** | 0.014 | 0.010 | -0.026 | 0.010 | 0.997 | — |  |  |  |
| **a diff** | -0.078 | 0.072 | -0.051 | 0.072 | 0.030 | -0.028 | — |  |  |
| **v diff** | -0.002 | 0.016 | -0.481 | 0.527 | 0.039 | 0.035 | 0.122 | — |  |
| **t0 diff** | 0.114 | -0.002 | 0.020 | -0.039 | -0.053 | 0.023 | -0.769 | -0.058 | — |

| **Table B8.** Product-moment correlation coefficients between the fitted parameters from EZ-diffusion fitting routine from Simulation 2 where drift rate (v) was manipulated between easy and hard conditions to have a medium effect size. | | | | | | | | | |
| --- | --- | --- | --- | --- | --- | --- | --- | --- | --- |
|  | **a (easy)** | **a (hard)** | **v**  **(easy)** | **v (hard)** | **t0 (easy)** | **t0 (hard)** | **a**  **diff** | **v**  **diff** | **t0**  **diff** |
| **a (easy)** | — |  |  |  |  |  |  |  |  |
| **a (hard)** | 0.980 | — |  |  |  |  |  |  |  |
| **v (easy)** | -0.043 | -0.036 | — |  |  |  |  |  |  |
| **v (hard)** | -0.042 | -0.038 | 0.496 | — |  |  |  |  |  |
| **t0 (easy)** | -0.003 | 0.015 | -0.011 | 0.023 | — |  |  |  |  |
| **t0 (hard)** | 0.009 | -0.001 | -0.021 | 0.018 | 0.980 | — |  |  |  |
| **a diff** | -0.082 | 0.118 | 0.031 | 0.020 | 0.088 | -0.049 | — |  |  |
| **v diff** | -0.001 | -0.003 | -0.482 | 0.522 | 0.034 | 0.040 | -0.011 | — |  |
| **t0 diff** | 0.060 | -0.078 | -0.051 | -0.021 | -0.123 | 0.077 | -0.686 | 0.029 | — |

**a Medium**

| **Table B9.** Product-moment correlation coefficients between the fitted parameters from fast-dm-30 fitting routine from Simulation 2 where boundary separation (a) was manipulated between easy and hard conditions to have a medium effect size. | | | | | | | | | |
| --- | --- | --- | --- | --- | --- | --- | --- | --- | --- |
|  | **a (easy)** | **a (hard)** | **v**  **(easy)** | **v (hard)** | **t0 (easy)** | **t0 (hard)** | **a**  **diff** | **v**  **diff** | **t0**  **diff** |
| **a (easy)** | — |  |  |  |  |  |  |  |  |
| **a (hard)** | 0.488 | — |  |  |  |  |  |  |  |
| **v (easy)** | 0.016 | -0.032 | — |  |  |  |  |  |  |
| **v (hard)** | 0.015 | -0.030 | 0.994 | — |  |  |  |  |  |
| **t0 (easy)** | 0.004 | -0.008 | -0.001 | -0.002 | — |  |  |  |  |
| **t0 (hard)** | 0.011 | -0.004 | 0.000 | -0.003 | 0.996 | — |  |  |  |
| **a diff** | -0.501 | 0.511 | -0.048 | -0.044 | -0.012 | -0.014 | — |  |  |
| **v diff** | -0.011 | 0.023 | -0.104 | 0.009 | -0.014 | -0.028 | 0.033 | — |  |
| **t0 diff** | 0.080 | 0.052 | 0.014 | -0.006 | -0.041 | 0.043 | -0.028 | -0.172 | — |

| **Table B10.** Product-moment correlation coefficients between the fitted parameters from EZ-diffusion fitting routine from Simulation 2 where boundary separation (a) was manipulated between easy and hard conditions to have a medium effect size. | | | | | | | | | |
| --- | --- | --- | --- | --- | --- | --- | --- | --- | --- |
|  | **a (easy)** | **a (hard)** | **v**  **(easy)** | **v (hard)** | **t0 (easy)** | **t0 (hard)** | **a**  **diff** | **v**  **diff** | **t0**  **diff** |
| **a (easy)** | — |  |  |  |  |  |  |  |  |
| **a (hard)** | 0.473 | — |  |  |  |  |  |  |  |
| **v (easy)** | 0.012 | -0.035 | — |  |  |  |  |  |  |
| **v (hard)** | 0.014 | -0.032 | 0.993 | — |  |  |  |  |  |
| **t0 (easy)** | -0.003 | -0.010 | -0.008 | -0.010 | — |  |  |  |  |
| **t0 (hard)** | 0.018 | -0.016 | -0.009 | -0.010 | 0.973 | — |  |  |  |
| **a diff** | -0.508 | 0.519 | -0.046 | -0.044 | -0.007 | -0.033 | — |  |  |
| **v diff** | 0.014 | 0.031 | -0.105 | 0.011 | -0.018 | -0.010 | 0.017 | — |  |
| **t0 diff** | 0.089 | -0.026 | -0.003 | 0.001 | -0.051 | 0.181 | -0.112 | 0.033 | — |

**t0 Medium**

| **Table B11.** Product-moment correlation coefficients between the fitted parameters from fast-dm-30 fitting routine from Simulation 2 where non-decision time (t0) was manipulated between easy and hard conditions to have a medium effect size. | | | | | | | | | |
| --- | --- | --- | --- | --- | --- | --- | --- | --- | --- |
|  | **a (easy)** | **a (hard)** | **v**  **(easy)** | **v (hard)** | **t0 (easy)** | **t0 (hard)** | **a**  **diff** | **v**  **diff** | **t0**  **diff** |
| **a (easy)** | — |  |  |  |  |  |  |  |  |
| **a (hard)** | 0.989 | — |  |  |  |  |  |  |  |
| **v (easy)** | 0.031 | 0.030 | — |  |  |  |  |  |  |
| **v (hard)** | 0.022 | 0.030 | 0.994 | — |  |  |  |  |  |
| **t0 (easy)** | 0.022 | 0.025 | 0.010 | 0.013 | — |  |  |  |  |
| **t0 (hard)** | 0.036 | 0.034 | 0.002 | 0.003 | 0.508 | — |  |  |  |
| **a diff** | -0.097 | 0.049 | -0.004 | 0.052 | 0.016 | -0.014 | — |  |  |
| **v diff** | -0.082 | -0.006 | -0.044 | 0.064 | 0.021 | 0.016 | 0.521 | — |  |
| **t0 diff** | 0.015 | 0.011 | -0.009 | -0.009 | -0.465 | 0.526 | -0.030 | -0.005 | — |

| **Table B12.** Product-moment correlation coefficients between the fitted parameters from EZ-diffusion fitting routine from Simulation 2 where non-decision time (t0) was manipulated between easy and hard conditions to have a medium effect size. | | | | | | | | | |
| --- | --- | --- | --- | --- | --- | --- | --- | --- | --- |
|  | **a (easy)** | **a (hard)** | **v**  **(easy)** | **v (hard)** | **t0 (easy)** | **t0 (hard)** | **a**  **diff** | **v**  **diff** | **t0**  **diff** |
| **a (easy)** | — |  |  |  |  |  |  |  |  |
| **a (hard)** | 0.983 | — |  |  |  |  |  |  |  |
| **v (easy)** | 0.022 | 0.028 | — |  |  |  |  |  |  |
| **v (hard)** | 0.015 | 0.026 | 0.994 | — |  |  |  |  |  |
| **t0 (easy)** | 0.003 | 0.011 | 0.016 | 0.019 | — |  |  |  |  |
| **t0 (hard)** | 0.026 | 0.014 | 0.004 | 0.008 | 0.497 | — |  |  |  |
| **a diff** | -0.094 | 0.091 | 0.033 | 0.061 | 0.045 | -0.064 | — |  |  |
| **v diff** | -0.067 | -0.020 | -0.050 | 0.060 | 0.023 | 0.043 | 0.253 | — |  |
| **t0 diff** | 0.024 | 0.004 | -0.012 | -0.010 | -0.464 | 0.537 | -0.110 | 0.021 | — |

**v Large**

| **Table B13.** Product-moment correlation coefficients between the fitted parameters from fast-dm-30 fitting routine from Simulation 2 where drift rate (v) was manipulated between easy and hard conditions to have a large effect size. | | | | | | | | | |
| --- | --- | --- | --- | --- | --- | --- | --- | --- | --- |
|  | **a (easy)** | **a (hard)** | **v**  **(easy)** | **v (hard)** | **t0 (easy)** | **t0 (hard)** | **a**  **diff** | **v**  **diff** | **t0**  **diff** |
| **a (easy)** | — |  |  |  |  |  |  |  |  |
| **a (hard)** | 0.985 | — |  |  |  |  |  |  |  |
| **v (easy)** | 0.089 | 0.076 | — |  |  |  |  |  |  |
| **v (hard)** | 0.041 | 0.049 | 0.490 | — |  |  |  |  |  |
| **t0 (easy)** | -0.055 | -0.049 | -0.053 | -0.048 | — |  |  |  |  |
| **t0 (hard)** | -0.041 | -0.047 | -0.052 | -0.051 | 0.996 | — |  |  |  |
| **a diff** | -0.178 | -0.003 | -0.082 | 0.041 | 0.041 | -0.030 | — |  |  |
| **v diff** | -0.051 | -0.030 | -0.538 | 0.471 | 0.007 | 0.004 | 0.123 | — |  |
| **t0 diff** | 0.165 | 0.022 | 0.010 | -0.033 | 0.003 | 0.089 | -0.819 | -0.042 | — |

| **Table B14.** Product-moment correlation coefficients between the fitted parameters from EZ-diffusion fitting routine from Simulation 2 where drift rate (v) was manipulated between easy and hard conditions to have a large effect size. | | | | | | | | | |
| --- | --- | --- | --- | --- | --- | --- | --- | --- | --- |
|  | **a (easy)** | **a (hard)** | **v**  **(easy)** | **v (hard)** | **t0 (easy)** | **t0 (hard)** | **a**  **diff** | **v**  **diff** | **t0**  **diff** |
| **a (easy)** | — |  |  |  |  |  |  |  |  |
| **a (hard)** | 0.971 | — |  |  |  |  |  |  |  |
| **v (easy)** | 0.061 | 0.069 | — |  |  |  |  |  |  |
| **v (hard)** | 0.031 | 0.046 | 0.492 | — |  |  |  |  |  |
| **t0 (easy)** | -0.044 | -0.045 | -0.046 | -0.044 | — |  |  |  |  |
| **t0 (hard)** | -0.028 | -0.063 | -0.052 | -0.049 | 0.981 | — |  |  |  |
| **a diff** | -0.090 | 0.149 | 0.035 | 0.063 | -0.003 | -0.148 | — |  |  |
| **v diff** | -0.032 | -0.025 | -0.532 | 0.476 | 0.003 | 0.005 | 0.027 | — |  |
| **t0 diff** | 0.081 | -0.097 | -0.038 | -0.028 | 0.016 | 0.212 | -0.744 | 0.011 | — |

**a Large**

| **Table B15.** Product-moment correlation coefficients between the fitted parameters from fast-dm-30 fitting routine from Simulation 2 where boundary separation (a) was manipulated between easy and hard conditions to have a large effect size. | | | | | | | | | |
| --- | --- | --- | --- | --- | --- | --- | --- | --- | --- |
|  | **a (easy)** | **a (hard)** | **v**  **(easy)** | **v (hard)** | **t0 (easy)** | **t0 (hard)** | **a**  **diff** | **v**  **diff** | **t0**  **diff** |
| **a (easy)** | — |  |  |  |  |  |  |  |  |
| **a (hard)** | 0.479 | — |  |  |  |  |  |  |  |
| **v (easy)** | 0.047 | 0.029 | — |  |  |  |  |  |  |
| **v (hard)** | 0.042 | 0.033 | 0.995 | — |  |  |  |  |  |
| **t0 (easy)** | -0.027 | 0.014 | -0.024 | -0.023 | — |  |  |  |  |
| **t0 (hard)** | -0.023 | 0.007 | -0.026 | -0.026 | 0.997 | — |  |  |  |
| **a diff** | -0.509 | 0.512 | -0.017 | -0.009 | 0.040 | 0.029 | — |  |  |
| **v diff** | -0.045 | 0.034 | -0.019 | 0.084 | 0.010 | -0.009 | 0.077 | — |  |
| **t0 diff** | 0.053 | -0.083 | -0.023 | -0.046 | -0.063 | 0.020 | -0.133 | -0.220 | — |

| **Table B16.** Product-moment correlation coefficients between the fitted parameters from EZ-diffusion fitting routine from Simulation 2 where boundary separation (a) was manipulated between easy and hard conditions to have a large effect size. | | | | | | | | | |
| --- | --- | --- | --- | --- | --- | --- | --- | --- | --- |
|  | **a (easy)** | **a (hard)** | **v**  **(easy)** | **v (hard)** | **t0 (easy)** | **t0 (hard)** | **a**  **diff** | **v**  **diff** | **t0**  **diff** |
| **a (easy)** | — |  |  |  |  |  |  |  |  |
| **a (hard)** | 0.473 | — |  |  |  |  |  |  |  |
| **v (easy)** | 0.038 | 0.011 | — |  |  |  |  |  |  |
| **v (hard)** | 0.034 | 0.009 | 0.994 | — |  |  |  |  |  |
| **t0 (easy)** | -0.040 | 0.012 | -0.015 | -0.016 | — |  |  |  |  |
| **t0 (hard)** | -0.005 | 0.012 | -0.024 | -0.026 | 0.972 | — |  |  |  |
| **a diff** | -0.530 | 0.497 | -0.027 | -0.024 | 0.050 | 0.016 | — |  |  |
| **v diff** | -0.045 | -0.020 | -0.049 | 0.058 | -0.016 | -0.019 | 0.026 | — |  |
| **t0 diff** | 0.146 | 0.000 | -0.040 | -0.041 | -0.116 | 0.123 | -0.144 | -0.010 | — |

**t0 Large**

| **Table B17.** Product-moment correlation coefficients between the fitted parameters from fast-dm-30 fitting routine from Simulation 2 where non-decision time (t0) was manipulated between easy and hard conditions to have a large effect size. | | | | | | | | | |
| --- | --- | --- | --- | --- | --- | --- | --- | --- | --- |
|  | **a (easy)** | **a (hard)** | **v**  **(easy)** | **v (hard)** | **t0 (easy)** | **t0 (hard)** | **a**  **diff** | **v**  **diff** | **t0**  **diff** |
| **a (easy)** | — |  |  |  |  |  |  |  |  |
| **a (hard)** | 0.991 | — |  |  |  |  |  |  |  |
| **v (easy)** | 0.006 | 0.011 | — |  |  |  |  |  |  |
| **v (hard)** | -0.001 | 0.011 | 0.994 | — |  |  |  |  |  |
| **t0 (easy)** | -0.019 | -0.017 | 0.024 | 0.027 | — |  |  |  |  |
| **t0 (hard)** | 0.012 | 0.010 | -0.003 | 0.002 | 0.483 | — |  |  |  |
| **a diff** | 0.002 | 0.134 | 0.042 | 0.092 | 0.015 | -0.009 | — |  |  |
| **v diff** | -0.065 | -0.004 | -0.047 | 0.062 | 0.030 | 0.048 | 0.455 | — |  |
| **t0 diff** | 0.030 | 0.027 | -0.026 | -0.024 | -0.509 | 0.508 | -0.023 | 0.049 | — |

| **Table B18.** Product-moment correlation coefficients between the fitted parameters from EZ-diffusion fitting routine from Simulation 2 where non-decision time (t0) was manipulated between easy and hard conditions to have a large effect size. | | | | | | | | | |
| --- | --- | --- | --- | --- | --- | --- | --- | --- | --- |
|  | **a (easy)** | **a (hard)** | **v**  **(easy)** | **v (hard)** | **t0 (easy)** | **t0 (hard)** | **a**  **diff** | **v**  **diff** | **t0**  **diff** |
| **a (easy)** | — |  |  |  |  |  |  |  |  |
| **a (hard)** | 0.981 | — |  |  |  |  |  |  |  |
| **v (easy)** | 0.011 | 0.005 | — |  |  |  |  |  |  |
| **v (hard)** | 0.004 | 0.003 | 0.993 | — |  |  |  |  |  |
| **t0 (easy)** | -0.024 | -0.006 | 0.011 | 0.012 | — |  |  |  |  |
| **t0 (hard)** | 0.006 | 0.001 | 0.002 | 0.009 | 0.472 | — |  |  |  |
| **a diff** | -0.060 | 0.134 | -0.033 | -0.005 | 0.091 | -0.025 | — |  |  |
| **v diff** | -0.064 | -0.017 | -0.055 | 0.061 | 0.013 | 0.063 | 0.244 | — |  |
| **t0 diff** | 0.029 | 0.007 | -0.009 | -0.003 | -0.518 | 0.509 | -0.113 | 0.049 | — |

**Simulation 3: Differences in All Parameters**

**All Small**

| **Table B19.** Product-moment correlation coefficients between the fitted parameters from fast-dm-30 fitting routine from Simulation 3 where all parameters were manipulated between easy and hard conditions to have a small effect size. | | | | | | | | | |
| --- | --- | --- | --- | --- | --- | --- | --- | --- | --- |
|  | **a (easy)** | **a (hard)** | **v**  **(easy)** | **v (hard)** | **t0 (easy)** | **t0 (hard)** | **a**  **diff** | **v**  **diff** | **t0**  **diff** |
| **a (easy)** | — |  |  |  |  |  |  |  |  |
| **a (hard)** | 0.514 | — |  |  |  |  |  |  |  |
| **v (easy)** | 0.045 | 0.022 | — |  |  |  |  |  |  |
| **v (hard)** | 0.037 | 0.075 | 0.483 | — |  |  |  |  |  |
| **t0 (easy)** | -0.061 | -0.009 | -0.005 | -0.005 | — |  |  |  |  |
| **t0 (hard)** | -0.041 | -0.093 | -0.037 | -0.027 | 0.504 | — |  |  |  |
| **a diff** | -0.518 | 0.467 | -0.025 | 0.036 | 0.054 | -0.051 | — |  |  |
| **v diff** | -0.007 | 0.053 | -0.490 | 0.526 | 0.000 | 0.009 | 0.060 | — |  |
| **t0 diff** | 0.019 | -0.086 | -0.033 | -0.023 | -0.483 | 0.513 | -0.105 | 0.009 | — |

| **Table B20.** Product-moment correlation coefficients between the fitted parameters from EZ-diffusion fitting routine from Simulation 3 where all parameters were manipulated between easy and hard conditions to have a small effect size. | | | | | | | | | |
| --- | --- | --- | --- | --- | --- | --- | --- | --- | --- |
|  | **a (easy)** | **a (hard)** | **v**  **(easy)** | **v (hard)** | **t0 (easy)** | **t0 (hard)** | **a**  **diff** | **v**  **diff** | **t0**  **diff** |
| **a (easy)** | — |  |  |  |  |  |  |  |  |
| **a (hard)** | 0.515 | — |  |  |  |  |  |  |  |
| **v (easy)** | 0.028 | 0.024 | — |  |  |  |  |  |  |
| **v (hard)** | 0.029 | 0.066 | 0.483 | — |  |  |  |  |  |
| **t0 (easy)** | -0.070 | -0.015 | 0.000 | -0.002 | — |  |  |  |  |
| **t0 (hard)** | -0.045 | -0.111 | -0.040 | -0.025 | 0.500 | — |  |  |  |
| **a diff** | -0.511 | 0.473 | -0.005 | 0.036 | 0.056 | -0.065 | — |  |  |
| **v diff** | 0.002 | 0.042 | -0.492 | 0.525 | -0.002 | 0.014 | 0.041 | — |  |
| **t0 diff** | 0.024 | -0.097 | -0.041 | -0.024 | -0.482 | 0.518 | -0.121 | 0.016 | — |

**All Medium**

| **Table B21.** Product-moment correlation coefficients between the fitted parameters from fast-dm-30 fitting routine from Simulation 3 where all parameters were manipulated between easy and hard conditions to have a medium effect size. | | | | | | | | | |
| --- | --- | --- | --- | --- | --- | --- | --- | --- | --- |
|  | **a (easy)** | **a (hard)** | **v**  **(easy)** | **v (hard)** | **t0 (easy)** | **t0 (hard)** | **a**  **diff** | **v**  **diff** | **t0**  **diff** |
| **a (easy)** | — |  |  |  |  |  |  |  |  |
| **a (hard)** | 0.512 | — |  |  |  |  |  |  |  |
| **v (easy)** | 0.021 | 0.003 | — |  |  |  |  |  |  |
| **v (hard)** | 0.072 | 0.098 | 0.488 | — |  |  |  |  |  |
| **t0 (easy)** | 0.045 | 0.027 | 0.045 | -0.002 | — |  |  |  |  |
| **t0 (hard)** | 0.010 | 0.007 | -0.025 | -0.056 | 0.480 | — |  |  |  |
| **a diff** | -0.511 | 0.477 | -0.019 | 0.025 | -0.019 | -0.003 | — |  |  |
| **v diff** | 0.049 | 0.093 | -0.518 | 0.494 | -0.047 | -0.030 | 0.043 | — |  |
| **t0 diff** | -0.034 | -0.019 | -0.069 | -0.053 | -0.515 | 0.506 | 0.016 | 0.017 | — |

| **Table B22.** Product-moment correlation coefficients between the fitted parameters from EZ-diffusion fitting routine from Simulation 3 where all parameters were manipulated between easy and hard conditions to have a medium effect size. | | | | | | | | | |
| --- | --- | --- | --- | --- | --- | --- | --- | --- | --- |
|  | **a (easy)** | **a (hard)** | **v**  **(easy)** | **v (hard)** | **t0 (easy)** | **t0 (hard)** | **a**  **diff** | **v**  **diff** | **t0**  **diff** |
| **a (easy)** | — |  |  |  |  |  |  |  |  |
| **a (hard)** | 0.500 | — |  |  |  |  |  |  |  |
| **v (easy)** | -0.003 | -0.015 | — |  |  |  |  |  |  |
| **v (hard)** | 0.062 | 0.068 | 0.487 | — |  |  |  |  |  |
| **t0 (easy)** | 0.043 | 0.025 | 0.050 | -0.002 | — |  |  |  |  |
| **t0 (hard)** | 0.024 | 0.002 | -0.021 | -0.049 | 0.475 | — |  |  |  |
| **a diff** | -0.525 | 0.475 | -0.012 | 0.004 | -0.019 | -0.023 | — |  |  |
| **v diff** | 0.063 | 0.082 | -0.519 | 0.494 | -0.052 | -0.027 | 0.016 | — |  |
| **t0 diff** | -0.018 | -0.023 | -0.069 | -0.046 | -0.512 | 0.513 | -0.004 | 0.024 | — |

**All Large**

| **Table B23.** Product-moment correlation coefficients between the fitted parameters from fast-dm-30 fitting routine from Simulation 3 where all parameters were manipulated between easy and hard conditions to have a large effect size. | | | | | | | | | |
| --- | --- | --- | --- | --- | --- | --- | --- | --- | --- |
|  | **a (easy)** | **a (hard)** | **v**  **(easy)** | **v (hard)** | **t0 (easy)** | **t0 (hard)** | **a**  **diff** | **v**  **diff** | **t0**  **diff** |
| **a (easy)** | — |  |  |  |  |  |  |  |  |
| **a (hard)** | 0.506 | — |  |  |  |  |  |  |  |
| **v (easy)** | 0.028 | -0.005 | — |  |  |  |  |  |  |
| **v (hard)** | 0.080 | 0.093 | 0.499 | — |  |  |  |  |  |
| **t0 (easy)** | 0.053 | 0.017 | 0.047 | -0.012 | — |  |  |  |  |
| **t0 (hard)** | 0.015 | 0.002 | -0.024 | -0.055 | 0.483 | — |  |  |  |
| **a diff** | -0.507 | 0.487 | -0.034 | 0.012 | -0.036 | -0.013 | — |  |  |
| **v diff** | 0.051 | 0.097 | -0.523 | 0.478 | -0.059 | -0.030 | 0.046 | — |  |
| **t0 diff** | -0.038 | -0.015 | -0.070 | -0.042 | -0.511 | 0.506 | 0.023 | 0.029 | — |

| **Table B24.** Product-moment correlation coefficients between the fitted parameters from EZ-diffusion fitting routine from Simulation 3 where all parameters were manipulated between easy and hard conditions to have a large effect size. | | | | | | | | | |
| --- | --- | --- | --- | --- | --- | --- | --- | --- | --- |
|  | **a (easy)** | **a (hard)** | **v**  **(easy)** | **v (hard)** | **t0 (easy)** | **t0 (hard)** | **a**  **diff** | **v**  **diff** | **t0**  **diff** |
| **a (easy)** | — |  |  |  |  |  |  |  |  |
| **a (hard)** | 0.489 | — |  |  |  |  |  |  |  |
| **v (easy)** | -0.005 | -0.028 | — |  |  |  |  |  |  |
| **v (hard)** | 0.060 | 0.053 | 0.495 | — |  |  |  |  |  |
| **t0 (easy)** | 0.039 | 0.014 | 0.053 | -0.009 | — |  |  |  |  |
| **t0 (hard)** | 0.019 | -0.018 | -0.011 | -0.037 | 0.476 | — |  |  |  |
| **a diff** | -0.519 | 0.492 | -0.022 | -0.008 | -0.024 | -0.037 | — |  |  |
| **v diff** | 0.064 | 0.080 | -0.525 | 0.480 | -0.062 | -0.025 | 0.014 | — |  |
| **t0 diff** | -0.019 | -0.032 | -0.062 | -0.028 | -0.508 | 0.516 | -0.013 | 0.036 | — |

**Appendix C—Examining the Impact of High Accuracy**

The analysis reported in this Appendix was inspired by a comment by a Reviewer^^[[1]](#footnote-1)^^ who suggested that a potential cause of the spurious correlation between *a*-difference and *t0*-difference could be the difficulty of diffusion models to accurately identify model parameters when accuracy is very high (as the parameter optimisation routine has few error trials to constrain parameter values); it might be then that the spurious correlation is caused (or exacerbated) by the presence of (real or simulated) participants with very high accuracy.

In an initial exploration of this, we re-analysed the data from Simulation 1 as suggested by the Reviewer. Specifically, we plotted the relationship between *a*-difference and *t0*-difference, but colour-coded individual data points by mean accuracy across “easy” and “hard” conditions (see Figure C1).


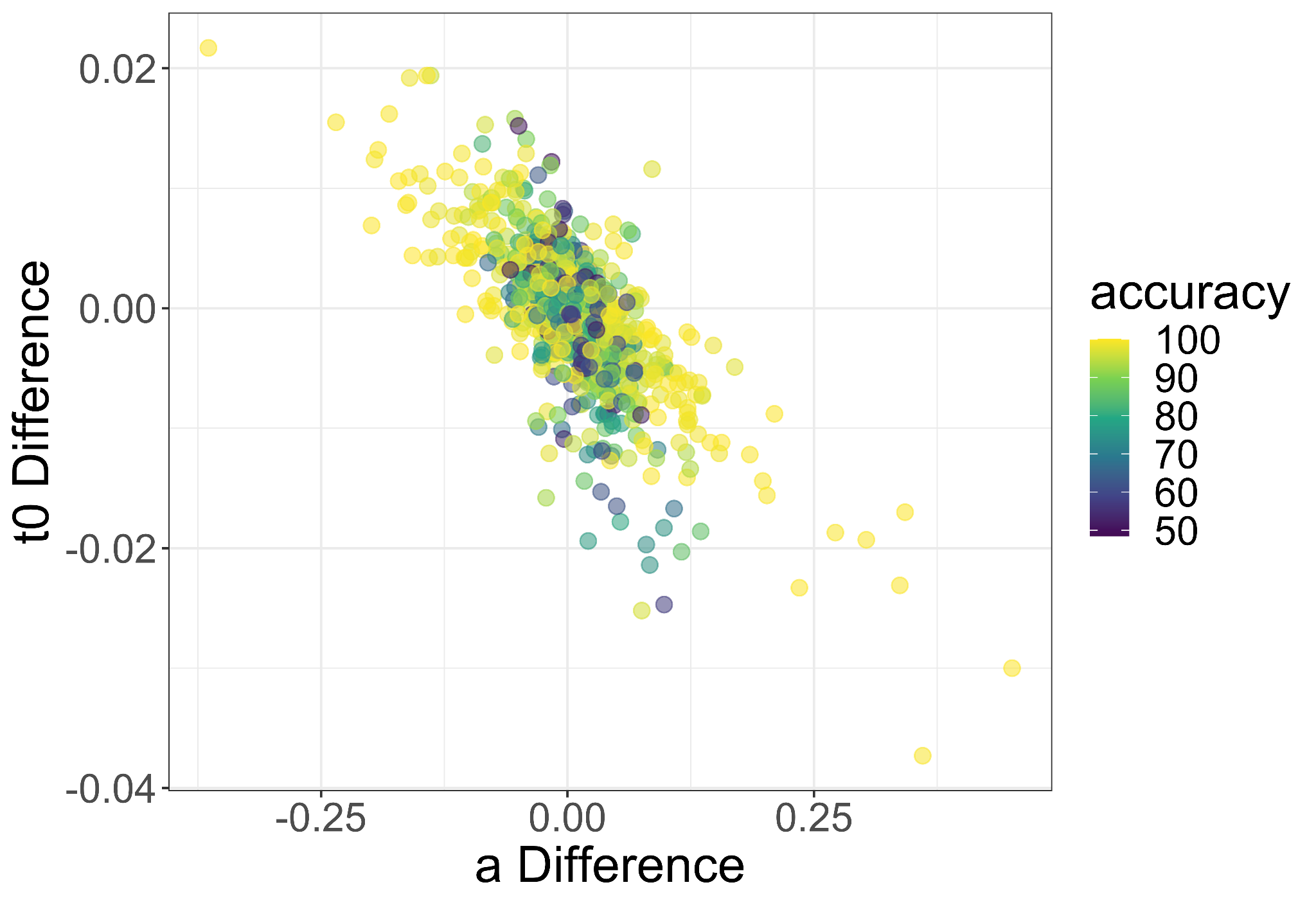


**Figure C1.** Scatterplot of the relationship between a-Difference and t0-Difference scores from Simulation 1, colour-coded by mean accuracy for each participant.

Consistent with the Reviewer’s observation, it does indeed appear that individuals with high levels of accuracy exhibit a strong negative correlation, whereas data points with lower accuracy tend to cluster more around the centre of the plot. We wished to therefore explore further the contribution of high accuracy performance to the occurrence of the spurious correlation reported in the main body of the paper.

**New Simulation**

In a first approach we conducted a new simulation where the model was forced to have lower accuracy performance. If the spurious correlation is caused by high accuracy rates causing issues with parameter identification (and hence, accurate parameter estimation), we should not observe the correlation when accuracy is forced to be lower. To achieve this, we repeated Simulation 1 but limited the random selection of the boundary separation parameter between 0.2–0.8 (in contrast to a range of 0.5–2.0). All other aspects of the simulation were identical to Simulation 1. The results of the correlation of interest—again colour-coded by accuracy as in Figure C1—are shown in Figure C2.

The mean accuracy rate across all participants in this simulation was 70.95%; only 0.4% of participants had a mean accuracy above 95%, and only 8.4% of participants had an accuracy above 90%. This suggests that the simulation was successful in reducing overall accuracy in simulated participants. Despite this lower accuracy, we still observed the strong negative correlation between *a*-difference and *t0*-difference, *r* = –.622.


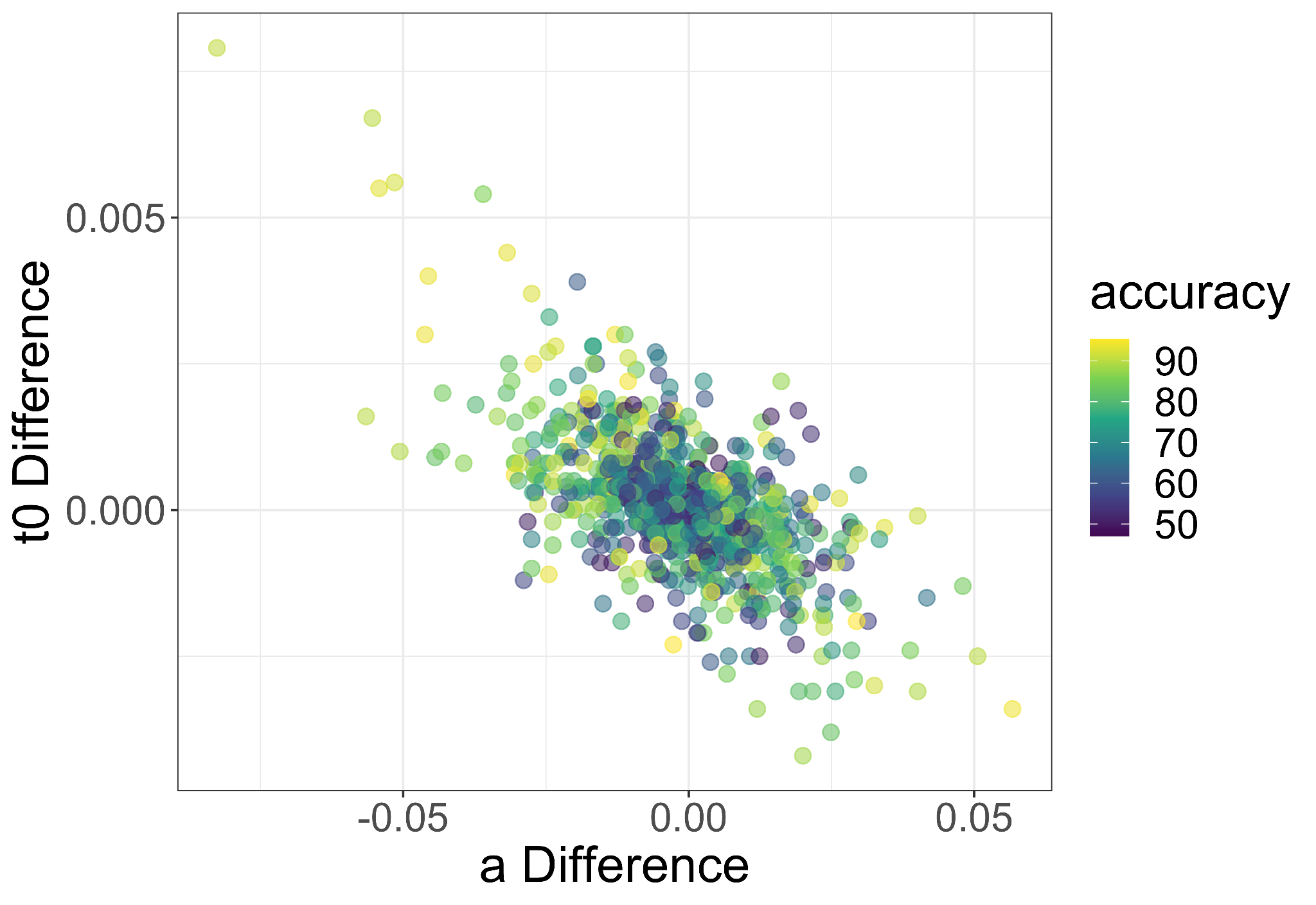


**Figure C2.** Scatterplot of the relationship between a-Difference and t0-Difference scores from the new Simulation of a model with enforced lower accuracy, colour-coded by mean accuracy for each participant.

**Quantile Split Re-Analysis of Simulations 1 and 4**

In a second step to investigate the impact of high accuracy, we re-analysed the data from Simulation 1 (DDM) and Simulation 4 (LBA) by splitting the 1,000 simulated participants from each simulation into four quantiles based on the mean accuracy per simulated participant (calculated as accuracy averaged across “easy” and “hard” conditions per simulated participant). The correlation between *a*-difference (*caution*-difference in the case of LBA) and *t0*-difference was then computed separately for the different quartiles (see Figures C3 and C4 for plots of these correlations). We observed that the correlation between *a*-difference and *t0*-difference occurred across all accuracy levels. In Simulation 1, the correlation amounted to *r* = –.85 in the quartile with highest accuracy, and *r* = –.69, *r* = –.69, *r* = –.66 in the other quartiles. In Simulation 4, the correlation was *r* = –.94 in the quartile with highest accuracy, and *r* = –.84, *r* = –.72, and *r* = –.78 in the other quartiles. Hence, in both simulations, the spurious correlation between the difference scores in non-decision time and response caution seemed to be more pronounced in the 25% of simulated subjects with highest overall accuracy. Importantly, though, the spurious correlation was observed across all accuracy ranges, even in the 25% artificial subjects with lowest accuracy, in both simulations. This suggests that high accuracy may inflate the spurious correlation, but it is not the only reason for the spurious correlation to occur.

The more pronounced correlation in the quartile with highest accuracy might be driven by the larger range of *a-*difference (*caution*-difference) scores in the 25% artificial participants with highest accuracy levels. One possible interpretation of this data pattern is that having very few error trials leads to less precise estimation of the boundary separation parameter, which in turn leads to a larger range of *a-*difference (*caution*-difference) scores, and therefore more pronounced correlations between *a-*difference and *t0*-difference scores.


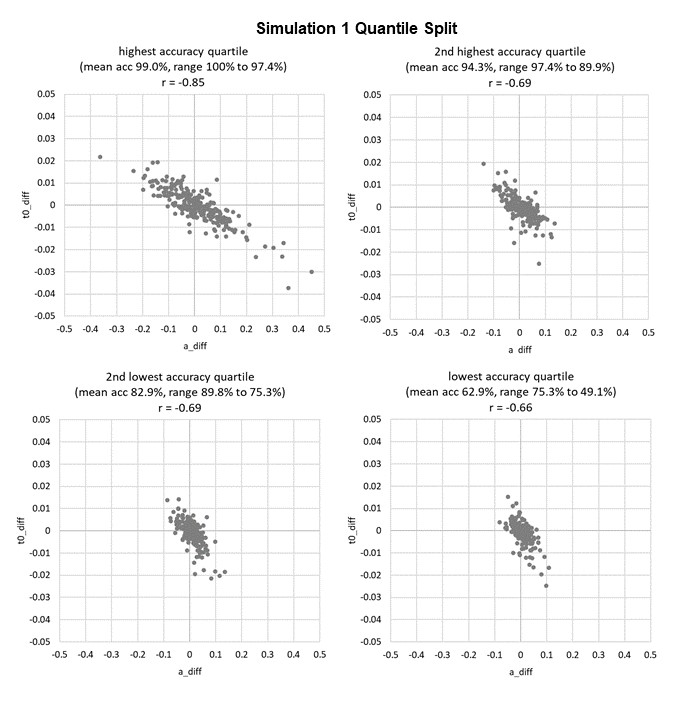
**Figure C3**. Quantile Split of Simulation 1 (DDM). Scatterplots of the relationship between *a*-difference (a_diff) and *t0*-difference (t0_diff) scores in the different accuracy quartiles (N=250 simulated participants per quartile). Points represent individual simulated participant difference scores in diffusion model parameters.


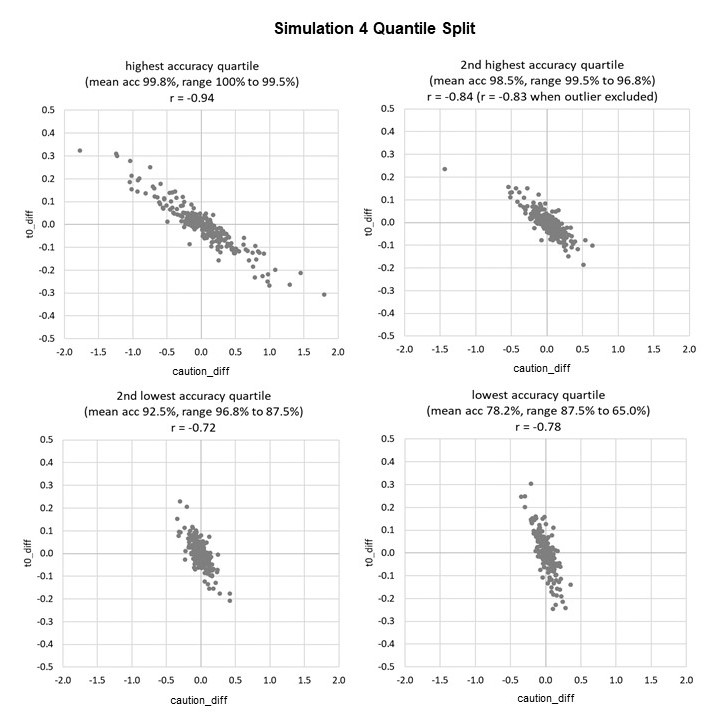


**Figure C4**. Quantile Split of Simulation 4 (LBA). Scatterplots of the relationship between *caution*-difference (caution_diff) and *t0*-difference (t0_diff) scores in the different accuracy quartiles (N=250 simulated participants per quartile). Points represent individual simulated participant difference scores in LBA model parameters. Note that the scales for the difference scores are different between Figures C3 and C4 due to the different parameterizations of the Drift-Diffusion Model (DDM) and the Linear Ballistic Accumulator Model (LBA) and/or different precision in parameter recovery in the two models.

To summarise, both the additional simulation with enforced lower accuracy, and the post-hoc analyses dividing the simulated participants by their accuracy level, suggest that high accuracy may inflate the spurious correlation between *t0*-difference and *a*-difference scores, but it is not the only reason for the spurious correlation to occur.

**Appendix D—Bayesian Hierarchical Diffusion Modelling**

We utilised the *hBayesDM* package (Ahn et al., 2017) in R to fit a Bayesian hierarchical diffusion model to the data from Experiment 1 of Dutilh et al. (2019). The model was fit to the “easy” and the “hard” condition separately using 2,000 samples (with 1,000 treated as warm-up samples, later discarded) from the posterior distribution across 4 chains. The default priors in the *hBayesDM* package were used, and initial parameter values for the sampling were determined randomly. The results of the fitting procedure are shown in Table B1. As can be seen, the large negative correlation between *a*-difference and *t0*-difference was also present in this fitting procedure (*r* = –.821).

| **Table D1.** Product-moment correlation coefficients between the fitted parameters from Bayesian hierarchical diffusion modelling of data from Experiment 1 of Dutilh et al. Diff = difference scores on parameters (hard minus easy). | | | | | | | | | |
| --- | --- | --- | --- | --- | --- | --- | --- | --- | --- |
|  | **a (easy)** | **a (hard)** | **v**  **(easy)** | **v (hard)** | **t0 (easy)** | **t0 (hard)** | **a**  **diff** | **v**  **diff** | **t0**  **diff** |
| **a (easy)** | — |  |  |  |  |  |  |  |  |
| **a (hard)** | 0.792 | — |  |  |  |  |  |  |  |
| **v (easy)** | 0.064 | 0.273 | — |  |  |  |  |  |  |
| **v (hard)** | -0.069 | 0.205 | 0.850 | — |  |  |  |  |  |
| **t0 (easy)** | -0.157 | 0.216 | 0.597 | 0.604 | — |  |  |  |  |
| **t0 (hard)** | -0.152 | -0.012 | 0.533 | 0.597 | 0.878 | — |  |  |  |
| **a diff** | -0.220 | 0.421 | 0.343 | 0.431 | 0.578 | 0.206 | — |  |  |
| **v diff** | -0.237 | -0.173 | -0.461 | 0.076 | -0.114 | -0.003 | 0.077 | — |  |
| **t0 diff** | 0.042 | -0.474 | -0.247 | -0.139 | -0.436 | 0.044 | -0.821 | 0.233 | — |

**Appendix E—Model Competition for Simulation 5**

We repeated the model competition analysis for the data generated in Simulation 5 with a “true” negative correlation between *a*-difference and *t0*-difference of *r* = –.70, but with no population-level difference between any of the main parameters between conditions. The details of the models’ specification that entered the competition are in Table E1, together with the fit statistics. As can be seen, the model where *v*, *a*, and *t0* are not free to vary across conditions is correctly selected as the winner (as this is the model that generated the data).

| **Table E1.** Results of the model competition approach to analysing data generated from Simulation 1. Each row depicts a different model based on whether drift rate (*v*), boundary separation (*a*), and non-decision time (*t0*) is free to vary across conditions or not (if yes, denoted by a tick, and by a cross if not). LL is the log-likelihood of the fit. AIC and BIC refer to the total Akaike’s and Bayesian Information Criteria, respectively, summed across all simulated participants. W_AIC_ and W_BIC_ represent Akaike weights for each model based on the AIC and BIC values, respectively. Bold & underlined model represents the winner of the model competition. | | | | | | | | |
| --- | --- | --- | --- | --- | --- | --- | --- | --- |
| **Model** | **Vary *v*?** | **Vary *a*?** | **Vary**  ***t0*?** | **LL** | **AIC** | **BIC** | **W_AIC_** | **W_BIC_** |
| **Model 1** | **x** | **x** | **x** | **-572,161** | **1,150,321** | **1,167,124** | **1** | **1** |
| Model 2 | ✓ | x | x | -576,991 | 1,161,981 | 1,184,385 | 0 | 0 |
| Model 3 | x | ✓ | x | -620,123 | 1,248,246 | 1,240,650 | 0 | 0 |
| Model 4 | x | x | ✓ | -707,278 | 1,422,557 | 1,444,960 | 0 | 0 |
| Model 5 | x | ✓ | ✓ | -716,287 | 1,442,574 | 1,470,579 | 0 | 0 |
| Model 6 | ✓ | x | ✓ | -708,398 | 1,426,797 | 1,454,801 | 0 | 0 |
| Model 7 | ✓ | ✓ | x | -621,822 | 1,253,644 | 1,281,649 | 0 | 0 |
| Model 8 | ✓ | ✓ | ✓ | -716,815 | 1,445,629 | 1,479,235 | 0 | 0 |

**Appendix F — Latent Change Score Modelling**

We explored whether the spurious correlation between *a*-difference and *t0*-difference also occurred when using latent change score modelling (Kievit et al., 2018; McArdle 2009; see Schubert et al., 2015 and 2022 for applications of this approach). To examine this, we ran a version of Simulation 1 reported in the main paper where there is no true difference between any of the diffusion model parameters between easy and hard conditions. Data were again simulated from 1,000 artificial participants in two experimental conditions using the same parameters as in Simulation 1. Differing from Simulation 1, we simulated 2,000 trials per condition per participant to reduce simulation noise even more (it was 1,000 trials per condition per participant in Simulation 1). The data for each condition for each participant were then separated into two halves (odd trials in one half, even trials in the other half). The diffusion model was then fit to each half separately. Within each half, *v*, *a*, and *t0* were allowed to vary freely across the easy and the hard conditions (i.e., the model fitting procedure within each half was identical to that in Simulation 1). Because odd trials and even trials were fitted separately, this procedure yielded (potentially) different parameter estimates for odd and even trials. The correlation matrices for odd and even trials are shown in Tables F1 and F2, respectively.

| **Table F1.** Product-moment correlation coefficients between the fitted parameters from the fast-dm-30 fitting routine for odd trials. Diff = difference scores on parameters (hard minus easy). | | | | | | | | | |
| --- | --- | --- | --- | --- | --- | --- | --- | --- | --- |
|  | **a (easy)** | **a (hard)** | **v**  **(easy)** | **v (hard)** | **t0 (easy)** | **t0 (hard)** | **a**  **diff** | **v**  **diff** | **t0**  **diff** |
| **a (easy)** | — |  |  |  |  |  |  |  |  |
| **a (hard)** | 0.990 | — |  |  |  |  |  |  |  |
| **v (easy)** | 0.045 | 0.045 | — |  |  |  |  |  |  |
| **v (hard)** | 0.038 | 0.047 | 0.994 | — |  |  |  |  |  |
| **t0 (easy)** | -0.047 | -0.042 | 0.000 | 0.003 | — |  |  |  |  |
| **t0 (hard)** | -0.043 | -0.046 | 0.003 | 0.003 | 0.997 | — |  |  |  |
| **a diff** | -0.040 | 0.100 | 0.005 | 0.063 | 0.034 | -0.020 | — |  |  |
| **v diff** | -0.060 | 0.012 | -0.040 | 0.073 | 0.020 | 0.002 | 0.512 | — |  |
| **t0 diff** | 0.055 | -0.049 | 0.036 | 0.007 | 0.012 | 0.084 | -0.741 | -0.251 | — |

| **Table F2.** Product-moment correlation coefficients between the fitted parameters from the fast-dm-30 fitting routine for even trials. Diff = difference scores on parameters (hard minus easy). | | | | | | | | | |
| --- | --- | --- | --- | --- | --- | --- | --- | --- | --- |
|  | **a (easy)** | **a (hard)** | **v**  **(easy)** | **v (hard)** | **t0 (easy)** | **t0 (hard)** | **a**  **diff** | **v**  **diff** | **t0**  **diff** |
| **a (easy)** | — |  |  |  |  |  |  |  |  |
| **a (hard)** | 0.990 | — |  |  |  |  |  |  |  |
| **v (easy)** | 0.046 | 0.037 | — |  |  |  |  |  |  |
| **v (hard)** | 0.044 | 0.042 | 0.994 | — |  |  |  |  |  |
| **t0 (easy)** | -0.044 | -0.043 | -0.001 | 0.003 | — |  |  |  |  |
| **t0 (hard)** | -0.043 | -0.050 | 0.002 | 0.004 | 0.997 | — |  |  |  |
| **a diff** | -0.017 | 0.123 | -0.063 | -0.011 | 0.003 | -0.051 | — |  |  |
| **v diff** | -0.015 | 0.052 | 0.006 | 0.114 | 0.033 | 0.017 | 0.476 | — |  |
| **t0 diff** | 0.007 | -0.098 | 0.042 | 0.018 | 0.001 | 0.074 | -0.751 | -0.216 | — |

**Latent Model Description**

We initially fitted the latent change score model as depicted in Figure F1. In this model, the best-fitting diffusion parameters are used as indicator variables to estimate latent variables for each of the three main diffusion model parameters in each condition. For example, the latent parameter reflecting boundary separation in the easy condition (i.e., a_easy) is estimated from the best-fitting diffusion model parameters for the boundary separation parameter in the easy condition for odd trials and for even trials (i.e., a_easy_odd & a_easy_even). Latent change scores for each diffusion model parameter—which reflect the difference in latent estimates of diffusion model parameters across easy and hard conditions—were estimated by regressing latent parameter estimates from the hard condition on the parameter estimates from the easy condition. The question of interest is then whether we observe a correlation between the latent difference score for boundary separation (shown as Δa) and non-decision time (Δt0).

It is possible that in the case of a spurious correlation, one would observe a large correlation between the two latent change scores, with at the same time very small variances, which could be an instance of a “Heywood case” (i.e., implausible or highly unlikely parameter estimates in factor analytic models; Heywood & Filon, 1931; see also Cooperman & Waller, 2021). If this were observed, the presumably spurious correlation should not be interpreted, and should not be used for further correlational analyses.

To fit the model we used the package *lavaan* in R (Rosseel, 2012). DDM parameters were standardised before fitting the structural equation model (SEM), and SEM optimisation was achieved via maximum likelihood.

**
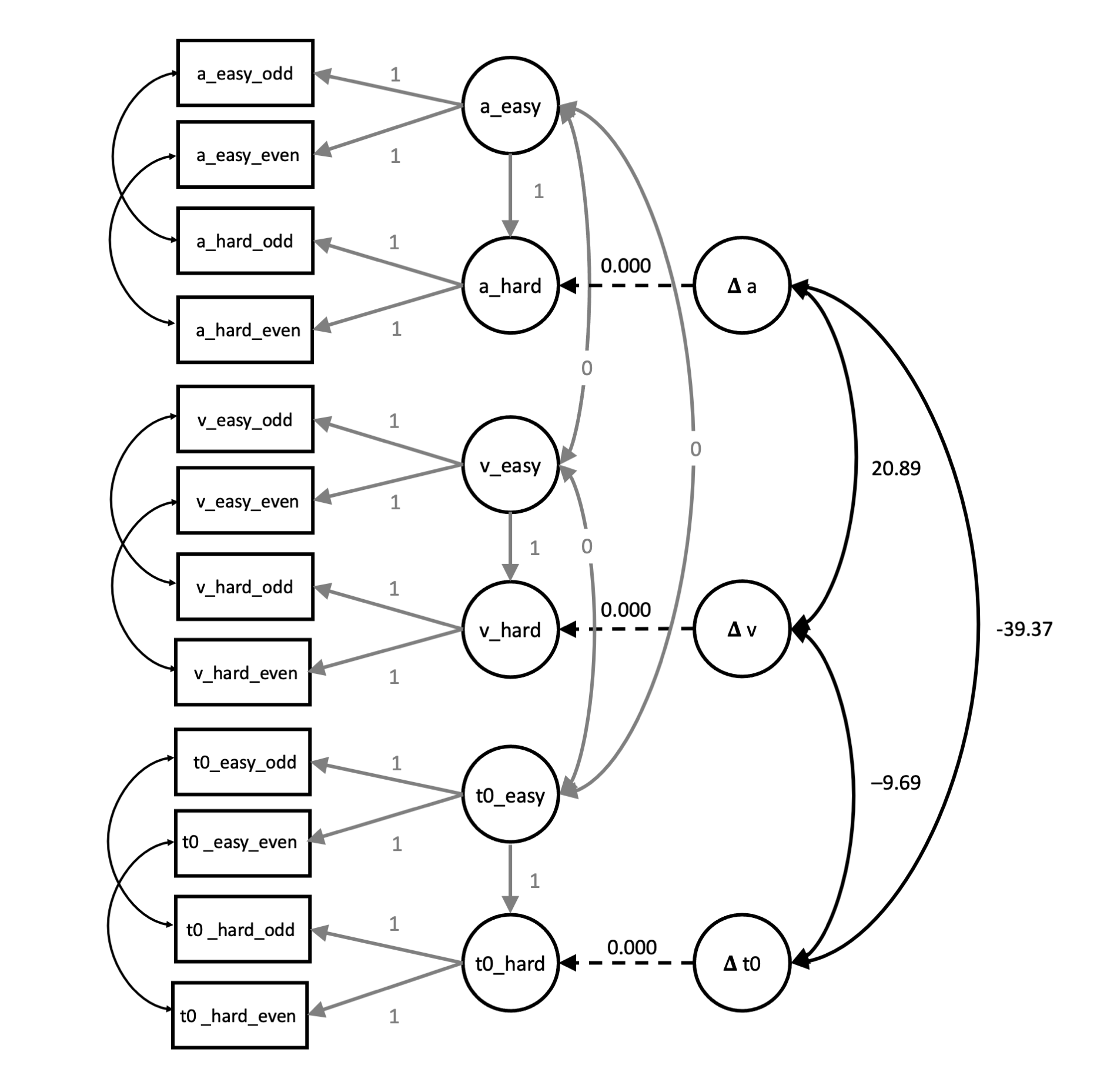
**

**Figure F1.** Path diagram for latent change score model 1. Rectangles represent indicator variables, and circles represent latent variables. Paths with thin lines to the left of the indicator variables reflect the residual covariances in the model. Grey path lines (and their numerical weights) indicate fixed paths; dashed lines indicate non-significant relationships.

The model demonstrated evidence of a Heywood case: The variances for the latent change scores were small and non-significant, yet the beta parameters reflecting the relationship between them were large. Note that the spurious negative relationship between *a*-difference and *t0*-difference was present (*b* = -39.37), as was the smaller positive relationship between *a*-difference and *v*-difference sometimes reported in the main body of the paper (*b* = 20.89). However, the latent change score model in Figure 1 did not provide a good fit to the data (see Table F3), but this lack of fit—and especially the significant result of the chi-square test—could be driven by the large sample size in the current simulation leading to small deviations of fit becoming highly significant due to extremely high power.

**Table F3.** Latent change score model fit indices. Np = number of parameters in each model. 𝝌^2^ = chi-square test (with * indicating significant tests). MSEA = Root mean square error of approximation. CFI = comparative fit index. AIC = Akaike’s Information Criterion. W_AIC_ = Akaike weights for each model based on the AIC values. Bold & underlined model represents the winner of the model competition.

| **Model** | **Np** | **𝝌^2^** | **RMSEA** | **CFI** | **AIC** | **W_AIC_** |
| --- | --- | --- | --- | --- | --- | --- |
| Model 1 | 27 | 2393.51* | 0.192 | 0.95 | -10,119 | 0.000 |
| Model 2 | 32 | 47.33 | 0.000 | 1.00 | -12,455 | 0.119 |
| **Model 3** | **30** | **47.38** | **0.000** | **1.00** | **-12,459** | **0.881** |

We then explored a second model (see Figure F2) where condition-specific correlations could occur between all diffusion model parameters in the indicator variables. Note that in Model 1 correlations were only allowed between matching DDM parameters across conditions (e.g., a_easy_odd was only allowed to correlate with a_hard_odd). These correlations between the fitted DDM parameters are very high (about *r* = .99, see Tables F1 and F2), because the data for the easy and hard conditions were generated using identical parameter values (see main text for details of Simulation 1). In contrast, the correlations between fitted DDM parameters within a condition (e.g., between a_easy_odd, v_easy_odd, and t0_easy_odd) were all very small (ranging from about *r* = .05 to *r* = –.05, see Tables F1 and F2), and were set to zero in Model 1. In Model 2, we set a different correlational structure between the indicator variables. In Model 2, DDM parameters could correlate with all other parameters in the same condition (e.g., now a_easy_odd could correlate with v_easy_odd and t0_easy_odd). The matching DDM parameters across conditions (e.g., between a_easy_odd and a_hard_odd) were no longer allowed to correlate in this model.^^[[2]](#footnote-2)^^


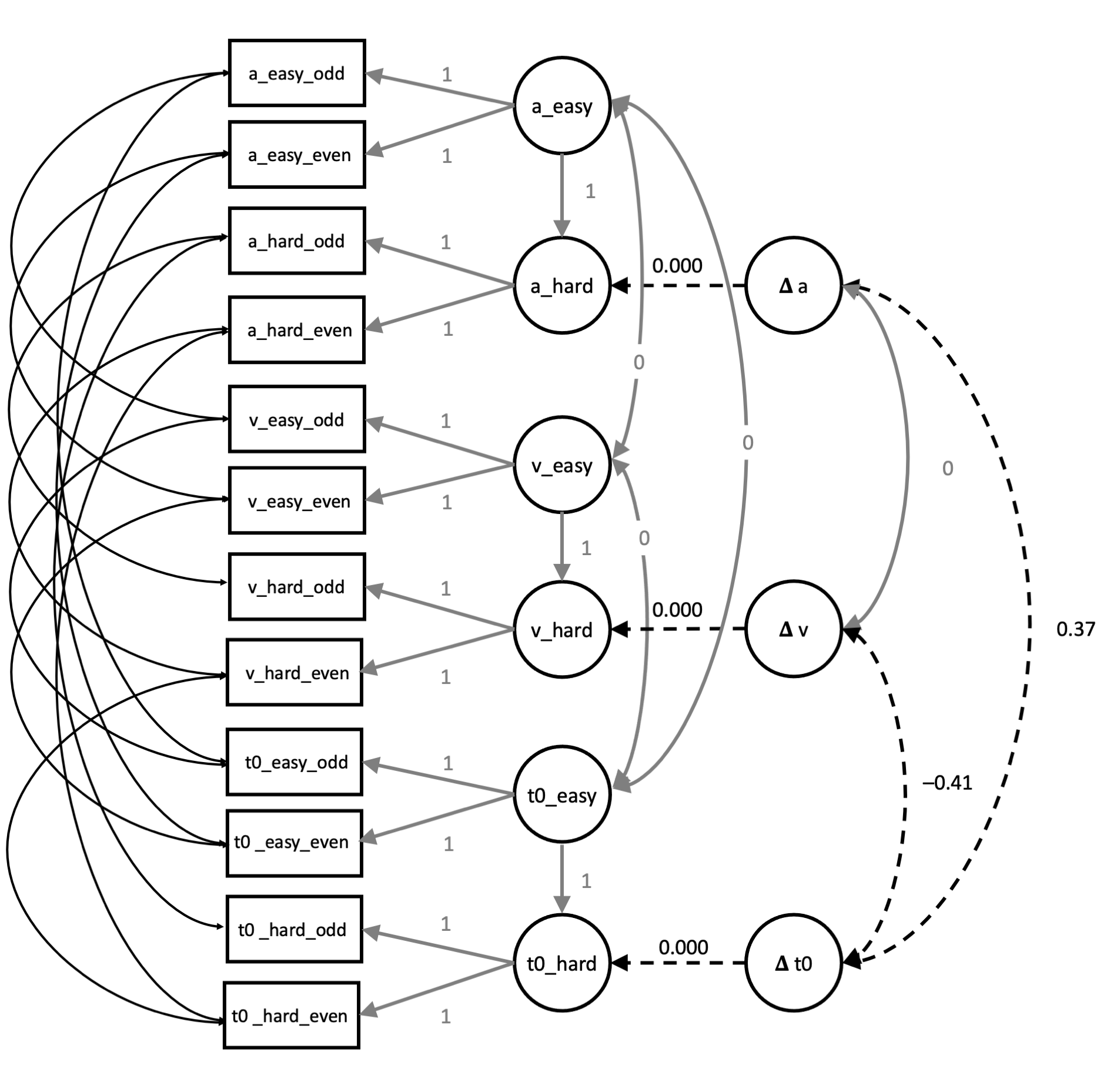


**Figure F2.** Path diagram for latent change score model 2. Rectangles represent indicator variables, and circles represent latent variables. Paths with thin lines to the left of the indicator variables reflect the residual covariances in the model. Grey path lines (and their numerical weights) indicate fixed paths; dashed lines indicate non-significant relationships.

Another change we made was to fix the correlation between the latent change scores *a*-difference and *v*-difference to zero^^[[3]](#footnote-3)^^. Model 2 showed a considerably better fit than Model 1 on all metrics (see Table F3). Critically, the variances of all latent change scores were still very close to zero and non-significant (as in Model 1), but now there were no significant relationships between *a*-difference and *t0*-difference, or between *v*-difference and *t0*-difference. This latter result was investigated by a third model (Model 3, see Figure F3) which fixed the relationships between all latent change scores to zero. As shown in Table F1, Model 3 was the best fit among all considered.

As described above, Models 2 and 3 differed from Model 1 with respect to the correlational structure between the indicator variables. In Models 2 and 3 (but not in Model 1), the DDM parameters within a condition (e.g., between a_easy_odd, v_easy_odd, and t0_easy_odd) were allowed to correlate. Interestingly, in Models 2 and 3, the estimated covariances between the DDM parameters within a condition were all significant, albeit they were very small in size.

This observation prompted us to inspect the within-condition correlations of parameters in our simulations more closely. We focused on all simulations in which we observed the spurious correlation between difference scores. On a descriptive level, we observed a consistent pattern in the fitted DDM parameters within a condition: The correlation between a and t0 is always slightly negative (around *r* = –0.05); the correlation between a and v is always slightly positive (around *r* = 0.05); the correlation between v and t0 is still smaller (around *r* = 0.005 or smaller). This pattern occurs in the simulation reported in Tables F1 and F2 in this Appendix, as well as in the simulations reported in Tables 4, 5, 7, 8, and 10 in the main text, and Tables B13 and B14 in Appendix B.

That is, in all the simulations where we find the pronounced negative correlation between boundary_difference and t0_difference (and in most cases, a moderate positive correlation between boundary_difference and v_difference), we find that within conditions, the correlation between a and t0 is always slightly negative (around *r* = –0.05), and the correlation between a and v is always slightly positive (around *r* = 0.05). In contrast, in the simulations where we do not observe the spurious correlations between difference scores, and in the empirical data, the pattern is less consistent (the empirical data are presumably noisier due to smaller sample size). It thus seems possible that these small but systematic interdependencies between the DDM parameters within a condition are somehow inflated when calculating difference scores.


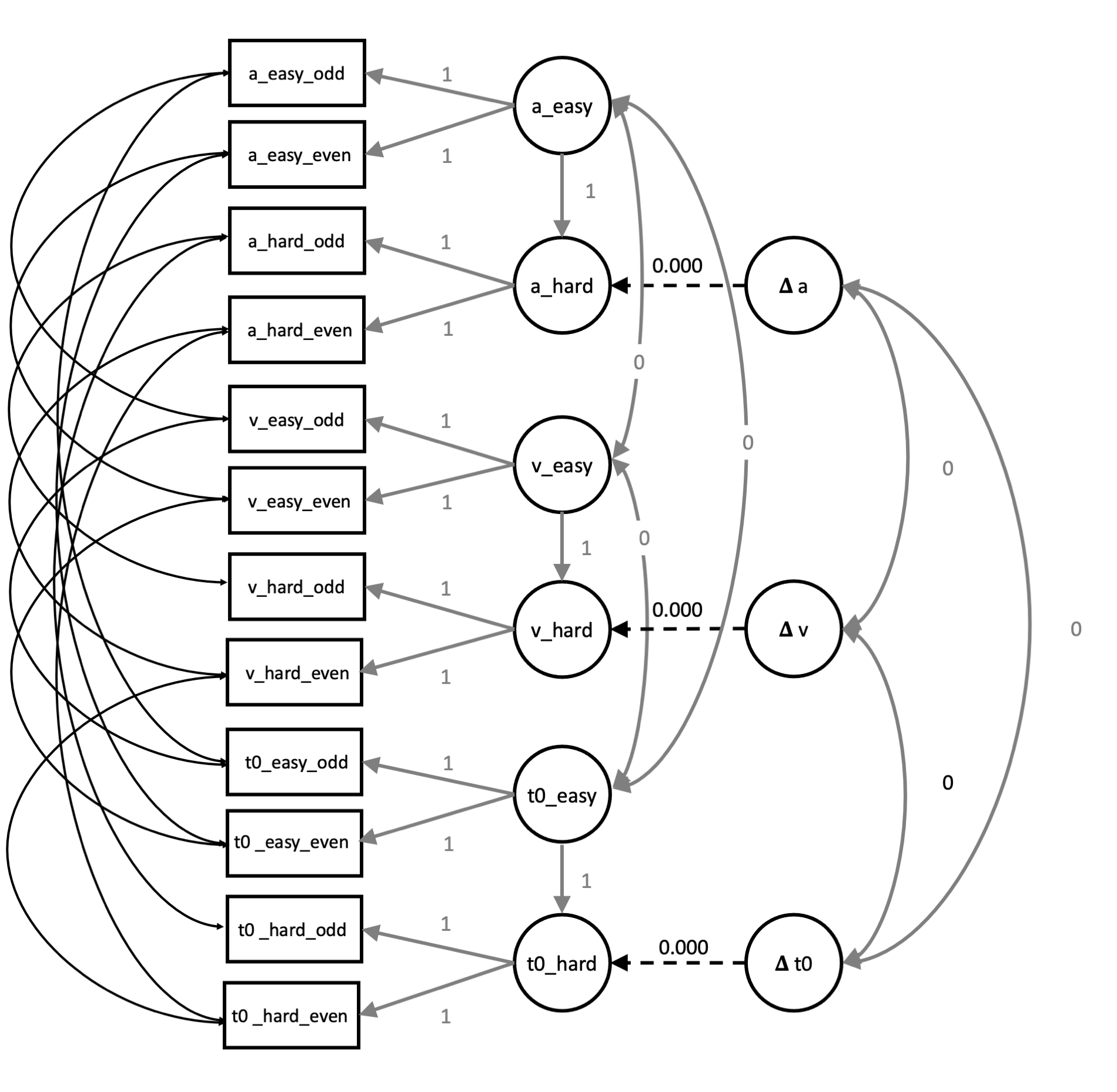


**Figure F3.** Path diagram for latent change score model 3. Rectangles represent indicator variables, and circles represent latent variables. Paths with thin lines to the left of the indicator variables reflect the residual covariances in the model. Grey path lines (and their numerical weights) indicate fixed paths; dashed lines indicate non-significant relationships.

**References**

Ahn W-Y, Haines N, & Zhang L. (2017). Revealing neurocomputational mechanisms of reinforcement learning and decision-making with the hBayesDM package. *Computational Psychiatry, 1,* 24–57. https://doi.org/10.1162/CPSY_a_00002.

Cooperman, A. W., & Waller, N. G. (2021). Heywood you go away! Examining causes, effects, and treatments for Heywood cases in exploratory factor analysis. *Psychological Methods*. Advance online publication. <https://doi.org/10.1037/met0000384>

Dutilh, G., Annis, J., Brown, S. D., Cassey, P., Evans, N. J., Grasman, R. P. P. P., Hawkins, G. E., Heathcote, A., Holmes, W. R., Krypotos, A.-M., Kupitz, C. N., Leite, F. P., Lerche, V., Lin, Y.-S., Logan, G. D., Palmeri, T. J., Starns, J. J., Trueblood, J. S., van Maanen, L., ... Donkin, C. (2019). The quality of response time data inference: A blinded, collaborative assessment of the validity of cognitive models. *Psychonomic Bulletin & Review*, *26*, 1051–1069. https://doi.org/10.3758/s13423-017-1417-2

Heywood, H. B. (1931). (Communicated by Filon, L. N. G.) On finite sequences of real numbers. *Proceedings of the Royal Society of London. Series A, Containing Papers of a Mathematical and Physical Character*, *134*, 486–501. <https://doi.org/10.1098/rspa.1931.0209>

Kievit, R. A., Brandmaier, A. M., Ziegler, G., van Harmelen, A.-L., de Mooij, S. M. M., Moutoussis, M., Goodyer, I. M., Bullmore, E., Jones, P. B., Fonagy, P., Lindenberger, U., & Dolan, R. J. (2018). Developmental cognitive neuroscience using latent change score models: A tutorial and applications. *Developmental Cognitive Neuroscience*, *33*, 99–117. https://doi.org/10.1016/j.dcn.2017.11.007

McArdle, J. J. (2009). Latent variable modeling of differences and changes with longitudinal data. *Annual Review of Psychology*, *60*, 577–605. https://doi.org/10.1146/annurev.psych.60.110707.163612

Rosseel, Y. (2012). *lavaan*: An R Package for Structural Equation Modeling. *Journal of Statistical Software*, *48*(2), 1–36. https://doi.org/10.18637/jss.v048.i02

Schubert, A.-L., Hagemann, D., Voss, A., Schankin, A., & Bergmann, K. (2015). Decomposing the relationship between mental speed and mental abilities. *Intelligence, 51*, 28-46. https://doi.org/10.1016/j.intell.2015.05.002

Schubert, A.-L., Löffler, C., & Hagemann, D. (2022). A neurocognitive psychometrics account of individual differences in attentional control. *Journal of Experimental Psychology: General*. Advance online publication. https://doi.org/10.1037/xge0001184

1. We are extremely grateful to Craig Hedge for this comment. [↑](#footnote-ref-1)
2. Whether the matching DDM parameters across conditions were allowed to correlate or not led to very similar model output in Models 2 and 3. [↑](#footnote-ref-2)
3. Note that we did try to fit a version of Model 2 where this correlation remained a free parameter, but the model fitting routine produced a warning that the covariance matrix of the latent variables was not positive definite. Ignoring this warning showed the relationship between a-difference and v-difference was non-significant. [↑](#footnote-ref-3)
